# Supplementary material for: A Rapamycin-Activated Caspase 9-Based Suicide Gene
Source: Mol Ther. 2018 Mar 9;26(5):1266–76. doi: 10.1016/j.ymthe.2018.03.001 (PMC5993966; doi:10.1016/j.ymthe.2018.03.001)

YMTHE, Volume *26*

Supplemental Information

A Rapamycin-Activated Caspase 9-Based Suicide Gene

Maria Stavrou, Brian Philip, Charlotte Traynor-White, Christopher G. Davis, Shimobi Onuoha, Shaun Cordoba, Simon Thomas, and Martin Pule

SUPPLEMENTARY FIGURE LEGENDS

Supplementary figure 1: Co-expressed FRB-Caspase9/FKBP-Caspase9 can be activated by Rapamycin to induce cell death

(a) Jurkats transduced with the FRB-Casp9/FKBP-Casp9 and the iCasp9 constructs were treated with increasing concentrations of Rapamycin or AP20187 for the Rapamycin induced constructs and the iCasp9 respectively. Cells were incubated for 24h and then cell ablation was assessed after Annexin V/7AAD staining by flow cytometry. FACS plots show the remaining cells expressing the constructs based on the eGFP/eBFP2 marker gene expression. The percentage of killing after drug administration is shown in (b). The percentage of killing was calculated based on the percentage of remaining live cells expressing the suicide construct after drug administration and upon normalisation to the respective untreated control. Results are from 4 independent experiments (n=4). Statistical analysis was performed using repeated measures two-way ANOVA with Sidak's post-test for multiple comparisons, **** indicates the significantly higher cell killing observed in the double transduced (FRB-Casp9/FKBP-Casp9) populations as opposed to the iCasp9 transduced cells. $P<0.0001$.

Supplementary figure 2: Comparison of rapaCasp9 variants with short L1 linker and L2 linkers of either 17 or 12 amino acids.

Functions of rapaCasp9 FRB-L1:5aa-FKBP12-L2:17aa-dCasp9 vs FRB-L1:5aa-FKBP12-L2:21aa-dCasp9 were compared in primary T-cells. Peripheral blood T-cells from 3 donors were transduced with each of the 2 constructs. FKBP-Casp9 transduced cells were used as negative control. Cells were treated with increasing concentrations of Rapamycin (0-100nM) and the % of killing was assessed as before after staining with 7-AAD/Annexin V and detecting the remaining GFP+ cells within the live cell population. Statistical Analysis was carried out using 2way Anova with Tukey's multiple comparison test.

Supplementary figure 3: Testing the impact of non-productive interaction on the function of the RapaCasp9 construct

RapaCasp9 was modified to contain the mutant FKBP (F36V) as used in the iCasp9 suicide gene. This change generates a mutant rapaCasp9 responsive to the homodimerizer drug (AP20187). Jurkat cells were transduced with rapaCasp9, mutant rapaCasp9, iCasp9 or FKBP-casp9 as negative control. Transduced Jurkats were treated with increasing concentrations of Rapamycin (rapaCasp9, FKBP-Casp9) or CID (mutant rapaCasp9, iCasp). Upon 24h incubation, the drug induced cell killing was assessed with Annexin V/7AAD staining by flow cytometry. Cell death was determined based on the remaining live cells expressing the constructs, as indicated by the eGFP marker gene expression. The percentage of killing was calculated based on the percentage of remaining eGFP+ live cells after drug treatment and upon normalisation to the untreated control for each condition. (a) Representative flow data showing eGFP expression vs forward-scatter area (FSC-A) is shown; (b) Results are from 2 independent experiments (n=2).

Supplementary figure 4: Function of flow-sorted rapaCasp9-CAR T-cells

(a) RQR8-CAR and RapaCasp9-CAR constructs used for T cell transduction. The CAR used comprises of the FMC63 scFv, the CD8 stalk and transmembrane domain and a 41BB-Z endodomain. Either RQR8 or rapaCasp9 were cloned in frame upstream from the CAR separated by a foot-and-mouth disease-like 2A peptide. T-cells transduced with either of the 2 CAR constructs were sorted for CAR^{high} expression and used in co-cultures with SupT1-CD19 target cells at a ratio 1:1 E:T in the absence of Rapamycin or with the addition of Rapamycin at 1nM. After 72h incubation, the cells were stained with 7-AAD viability dye to gate to the viable cell population and CD3 for the discrimination of the T cells (CD3+) from the target cells (CD3-) and were analyzed by flow-cytometry. (b) Plots representative of a single experiment are shown. Target cell and CAR T-cell gates are indicated on the plots. (c) The percentage of viable target cells after 72h co-culture with the indicated T-cell populations is shown. Data is collective from 4 separate experiments. The percentage of remaining viable target cells was determined after normalising to the number of target cells recovered from co-cultures carried out with NT T-cells in the absence of the drug. Lines indicate the mean value of each condition for 4 separate donors. Statistical analysis was carried out using repeated measures two-way ANOVA with Sidak's post-test for multiple comparisons, ****P<0.0001.

Supplementary figure 5: Control target cell killing and Rapamycin effect after activation

(a) CD19 negative target cell control for figure 4(f) is shown. QBEND/10 sorted RapaCasp9-Q8-CAR T-cells or RQR8-CAR T-cells were incubated without Rapamycin or with increasing concentrations of Rapamycin and challenged with SupT1 cells (which are CD19 negative). (b) To test the ability of Rapamycin to kill rapaCasp9-CAR T-cells after CAR activation, RQR8-CAR or rapaCasp9-CAR T-cells were transduced and sorted. These T cells were used in co-cultures with SupT1-CD19 target cells. Co-cultures were set up at E:T ratio 1:1 with $5X10^4$ targets. Co-cultures were kept in the absence of Rapamycin for 24h-to allow the stimulation of the T cells by the targets. After 24h incubation, cells were treated with 1nM Rapamycin or were left untreated. The % of remaining T cells was assessed after FACS analysis 3d post administration of the drug. Almost complete ablation of T cells is observed within the populations expressing the rapaCasp9-CAR when they are cultured in the presence of Rapamycin. Statistical analysis was carried out using repeated measures two-way ANOVA with Sidak's post-test for multiple comparisons, ****P<0.0001.

Supplementary figure 6: Proliferation, killing and phenotype of rapaCasp9-CAR T-cells

Proliferation: NT T-cells or QBEND/10 sorted T-Cells expressing either RQR8-CAR or rapaCasp9-CAR were labelled with Cell Trace Violet and then used in 1:1 co-cultures with different target cell lines namely SupT1, SupT1.CD19, Raji and Nalm6 cells. Cell proliferation was assessed 4 days later by FACS analysis based on the observed dilution of the Cell Trace Violet Dye. NT T cells included as control to define the basal level of T cells proliferation. (a) Representative flow plots from one donor are shown; (b) The percentage of proliferating cells in each co-culture for the NT cells is shown. No difference between RQR8 and rapaCasp9 CAR T-cells was observed. Killing: NT T-cells or QBEND/10 sorted T-cells expressing either RQR8-CAR or rapaCasp9-CAR were labeled with cell trace violet and co-cultured 1:1 with either SupT1, SupT1.CD19, Raji or Nalm6 cells for 24 hours. (c) Flow-cytometric analysis of live cells corrected

for bead counting allowed the determination of surviving target cells. Cumulative data from 3 donors normalized against counts from NT effector cells are shown. There is no difference between RQR8-CAR or rapaCasp9 CAR T-cells. Phenotype: NT or sorted QBEND/10 sorted memory phenotype was determined based on the expression of CD62L and CD45RA at different time points after their transduction. (d) The memory phenotype of the transduced populations on day 7 post transduction and upon sorting was determined. (i) Cumulative data from 3 donors for CD4+ cells is shown; (ii) for CD8+ cells; (iii) shows the % of CD4+ and CD8+ cells within the transduced populations. Statistical analysis was carried out using an unpaired parametric T test and showed no difference between RQR8- and rapaCasp9-CAR T-cells.

AMINO ACID SEQUENCES

Sequences of all open reading frames (ORF) of the plasmids used in the study

>FKBP-caspase 9

MLEGVQVETISPGDGRTFPKRGQTCVVHYTGMLEDGKKFDSSRDRNKPFKFMLGKQEVIRGWEEGVAQMS
VGQRAKLTISPDYAYGATGHPGIIPPHATLVFDVELLKLESGGGSGVDGFGDVGALESLRGNADLAYILS
MEPCGHCLIINNVNFCRESGLRTRTGSNIDCEKLRRRFSSLHFMVEVKGDLTAKKMVLALLELAQQDHGA
LDCCVVVILSHGCQASHLQFPGAVYGTDGCPVSVEKIVNIFNGTSCPSLGGKPKLFFIQACGGEQKDHGF
EVASTSPEDESPGSNPEPDATPFQEGLRTFDQLDAISSLPTPSDIFVSYSTFPGFVSWRDPKSGSWYVET
LDDIFEQWAHSEDLQSLLLRVANAVSVKGIYKQMPGCFNFLRKKLFFKTSAS*

>FRB-caspase 9

MASRILWHEMWHEGLEEASRLYFGERNVKGMFEVLEPLHAMMERGPQTLKETSFNQAYGRDLMEAQEWCR
KYMKSGNVKDLLQAWDLYYHVFRRISKLEYSGGGSGVDGFGDVGALESLRGNADLAYILSMEPCGHCLII
NNVNFCRESGLRTRTGSNIDCEKLRRRFSSLHFMVEVKGDLTAKKMVLALLELAQQDHGALDCCVVVILS
HGCQASHLQFPGAVYGTDGCPVSVEKIVNIFNGTSCPSLGGKPKLFFIQACGGEQKDHGFEVASTSPEDE
SPGSNPEPDATPFQEGLRTFDQLDAISSLPTPSDIFVSYSTFPGFVSWRDPKSGSWYVETLDDIFEQWAH
SEDLQSLLLRVANAVSVKGIYKQMPGCFNFLRKKLFFKTSAS*

>rapaCasp9 (FRB-L5aa-FKBP-L17aa-caspase9)

MASRILWHEMWHEGLEEASRLYFGERNVKGMFEVLEPLHAMMERGPQTLKETSFNQAYGRDLMEAQEWCR
KYMKSGNVKDLLQAWDLYYHVFRRISKLEYSGGGSLEGVQVETISPGDGRTFPKRGQTCVVHYTGMLEDG
KKFDSSRDRNKPFKFMLGKQEVIRGWEEGVAQMSVGQRAKLTISPDYAYGATGHPGIIPPHATLVFDVEL
LKLESGGGGSGGGGSGGGGSGVDGFGDVGALESLRGNADLAYILSMEPCGHCLIINNVNFCRESGLRTRT
GSNIDCEKLRRRFSSLHFMVEVKGDLTAKKMVLALLELAQQDHGALDCCVVVILSHGCQASHLQFPGAVY
GTDGCPVSVEKIVNIFNGTSCPSLGGKPKLFFIQACGGEQKDHGFEVASTSPEDESPGSNPEPDATPFQE
GLRTFDQLDAISSLPTPSDIFVSYSTFPGFVSWRDPKSGSWYVETLDDIFEQWAHSEDLQSLLLRVANAV
SVKGIYKQMPGCFNFLRKKLFFKTSAS*

>FRB-casp9-FKBP

MASRILWHEMWHEGLEEASRLYFGERNVKGMFEVLEPLHAMMERGPQTLKETSFNQAYGRDLMEAQEWCR
KYMKSGNVKDLLQAWDLYYHVFRRISKLEYSGGGGSGGGGSGGGGSGVDGFGDVGALESLRGNADLAYIL
SMEPCGHCLIINNVNFCRESGLRTRTGSNIDCEKLRRRFSSLHFMVEVKGDLTAKKMVLALLELAQQDHG
ALDCCVVVILSHGCQASHLQFPGAVYGTDGCPVSVEKIVNIFNGTSCPSLGGKPKLFFIQACGGEQKDHG
FEVASTSPEDESPGSNPEPDATPFQEGLRTFDQLDAISSLPTPSDIFVSYSTFPGFVSWRDPKSGSWYVE
TLDDIFEQWAHSEDLQSLLLRVANAVSVKGIYKQMPGCFNFLRKKLFFKTSASGGGGSGGGGSGGGGSLE
GVQVETISPGDGRTFPKRGQTCVVHYTGMLEDGKKFDSSRDRNKPFKFMLGKQEVIRGWEEGVAQMSVGQ
RAKLTISPDYAYGATGHPGIIPPHATLVFDVELLKLES*

>FKBP-casp9-FRB

```
MLEGVQVETISPGDGRTFPKRGQTCVVHYTGMLEDGKKFDSSRDRNKPFKFMLGKQEVIRGWEEGVAQMS
VGQRAKLTISPDYAYGATGHPGIIPPHATLVFDVELLKLESGGGGSGGGGSGGGGSGVDGFGDVGALESL
RGNADLAYILSMEPCGHCLIINNVNFCRESGLRTRTGSNIDCEKLRRRFSSLHFMVEVKGDLTAKKMVLA
LLELAQQDHGALDCCVVVILSHGCQASHLQFPGAVYGTDGCPVSVEKIVNIFNGTSCPSLGGKPKLFFIQ
ACGGEQKDHGFEVASTSPEDESPGSNPEPDATPFQEGLRTFDQLDAISSLPTPSDIFVSYSTFPGFVSWR
DPKSGSWYVETLDDIFEQWAHSEDLQSLLLRVANAVSVKGIYKQMPGCFNFLRKKLFFKTSASGGGGSGG
GGSGGGGSMASRILWHEMWHEGLEEASRLYFGERNVKGMFEVLEPLHAMMERGPQTLKETSFNQAYGRDL
MEAQEWCRKYMKSGNVKDLLQAWDLYYHVFRRISKLEY*
```

>FKBP-casp9-2A-FRB-FRBw

```
MLEGVQVETISPGDGRTFPKRGQTCVVHYTGMLEDGKKFDSSRDRNKPFKFMLGKQEVIRGWEEGVAQMS
VGQRAKLTISPDYAYGATGHPGIIPPHATLVFDVELLKLESGGGSGVDGFGDVGALESLRGNADLAYILS
MEPCGHCLIINNVNFCRESGLRTRTGSNIDCEKLRRRFSSLHFMVEVKGDLTAKKMVLALLELAQQDHGA
LDCCVVVILSHGCQASHLQFPGAVYGTDGCPVSVEKIVNIFNGTSCPSLGGKPKLFFIQACGGEQKDHGF
EVASTSPEDESPGSNPEPDATPFQEGLRTFDQLDAISSLPTPSDIFVSYSTFPGFVSWRDPKSGSWYVET
LDDIFEQWAHSEDLQSLLLRVANAVSVKGIYKQMPGCFNFLRKKLFFKTSASQCTNYALLKLAGDVESNP
GPGVQVETISPGDGRTFPKRGQTCVVHYTGMLEDGKKVDSSRDRNKPFKFMLGKQEVIRGWEEGVAQMSV
GQRAKLTISPDYAYGATGHPGIIPPHATLVFDVELLKLESGGGGSGGGGSMLEGVQVETISPGDGRTFPK
RGQTCVVHYTGMLEDGKKFDSSRDRNKPFKFMLGKQEVIRGWEEGVAQMSVGQRAKLTISPDYAYGATGH
PGIIPPHATLVFDVELLKLES*
```

>Casp9-FRB-FKBP

```
MVDGFGDVGALESLRGNADLAYILSMEPCGHCLIINNVNFCRESGLRTRTGSNIDCEKLRRRFSSLHFMV
EVKGDLTAKKMVLALLELAQQDHGALDCCVVVILSHGCQASHLQFPGAVYGTDGCPVSVEKIVNIFNGTS
CPSLGGKPKLFFIQACGGEQKDHGFEVASTSPEDESPGSNPEPDATPFQEGLRTFDQLDAISSLPTPSDI
FVSYSTFPGFVSWRDPKSGSWYVETLDDIFEQWAHSEDLQSLLLRVANAVSVKGIYKQMPGCFNFLRKKL
FFKTSASGGGGSGGGGSGGGGSGASRILWHEMWHEGLEEASRLYFGERNVKGMFEVLEPLHAMMERGPQT
LKETSFNQAYGRDLMEAQEWCRKYMKSGNVKDLLQAWDLYYHVFRRISKLEYSGGGSLEGVQVETISPGD
GRTFPKRGQTCVVHYTGMLEDGKKFDSSRDRNKPFKFMLGKQEVIRGWEEGVAQMSVGQRAKLTISPDYA
YGATGHPGIIPPHATLVFDVELLKLESGGGG*
```

>iCasp9

```
MLEGVQVETISPGDGRTFPKRGQTCVVHYTGMLEDGKKVDSSRDRNKPFKFMLGKQEVIRGWEEGVAQMS
VGQRAKLTISPDYAYGATGHPGIIPPHATLVFDVELLKLESGGGSGVDGFGDVGALESLRGNADLAYILS
MEPCGHCLIINNVNFCRESGLRTRTGSNIDCEKLRRRFSSLHFMVEVKGDLTAKKMVLALLELAQQDHGA
LDCCVVVILSHGCQASHLQFPGAVYGTDGCPVSVEKIVNIFNGTSCPSLGGKPKLFFIQACGGEQKDHGF
EVASTSPEDESPGSNPEPDATPFQEGLRTFDQLDAISSLPTPSDIFVSYSTFPGFVSWRDPKSGSWYVET
LDDIFEQWAHSEDLQSLLLRVANAVSVKGIYKQMPGCFNFLRKKLFFKTSASRAEGRGSLLTCGDVEENP
GPMGLVRRGARAGPRMPRGWTALCLLSLLPSGFMAELPTQGTFSNVSTNVSPAKPTTTPAPRPPTPAPTI
```

ASQPLSLRPEACRPAAGGAVHTRGLDFACDIYIWAPLAGTCGVLLLSLVITLYCNHRNRRRVCKCPRPVV
*

>FMC63-CAR (Campana)

MGTSLLCWMALCLLGADHADACPYSNPSLCSGGGGSELPTQGTFSNVSTNVSPAKPTTTACPYSNPSLCS
GGGGSPAPRPPTPAPTIASQPLSLRPEACRPAAGGAVHTRGLDFACDIYIWAPLAGTCGVLLLSLVITLY
CNHRNRRRVCKCPRPVVRAEGRGSLLTCGDVEENPGPMETDTLLLWVLLLWVPGSTGDIQMTQTTSSLSA
SLGDRVTISCRASQDISKYLNWYQQKPDGTVKLLIYHTSRLHSGVPSRFSGSGSGTDYSLTISNLEQEDI
ATYFCQQGNTLPYTFGGGTKLEITKAGGGGSGGGGSGGGGSGGGGSEVKLQESGPGLVAPSQSLSVTCTV
SGVSLPDYGVSWIRQPPRKGLEWLGVIWGSETTYYNSALKSRLTIIKDNSKSQVFLKMNSLQTDDTAIYY
CAKHYYYGGSYAMDYWGQGTSVTVSSDPTTTPAPRPPTPAPTIASQPLSLRPEACRPAAGGAVHTRGLDF
ACDIYIWAPLAGTCGVLLLSLVITLYCKRGRKKLLYIFKQPFMRPVQTTQEEDGCSCRFPEEEEGGCELR
VKFSRSADAPAYQQGQNQLYNELNLGRREEYDVLDKRRGRDPEMGGKPRRKNPQEGLYNELQKDKMAEAY
SEIGMKGERRRGKGHDGLYQGLSTATKDTYDALHMQALPPR*

>rapaCasp9-CAR

MASRILWHEMWHEGLEEASRLYFGERNVKGMFEVLEPLHAMMERGPQTLKETSFNQAYGRDLMEAQEWCR
KYMKSGNVKDLLQAWDLYYHVFRRISKLEYSGGGSLEGVQVETISPGDGRTFPKRGQTCVVHYTGMLEDG
KKFDSSRDRNKPFKFMLGKQEVIRGWEEGVAQMSVGQRAKLTISPDYAYGATGHPGIIPPHATLVFDVEL
LKLESGGGGSGGGGSGGGGSGVDGFGDVGALESLRGNADLAYILSMEPCGHCLIINNVNFCRESGLRTRT
GSNIDCEKLRRRFSSLHFMVEVKGDLTAKKMVLALLELAQQDHGALDCCVVVILSHGCQASHLQFPGAVY
GTDGCPVSVEKIVNIFNGTSCPSLGGKPKLFFIQACGGEQKDHGFEVASTSPEDESPGSNPEPDATPFQE
GLRTFDQLDAISSLPTPSDIFVSYSTFPGFVSWRDPKSGSWYVETLDDIFEQWAHSEDLQSLLLRVANAV
SVKGIYKQMPGCFNFLRKKLFFKTSASRAEGRGSLLTCGDVEENPGPMETDTLLLWVLLLWVPGSTGDIQ
MTQTTSSLSASLGDRVTISCRASQDISKYLNWYQQKPDGTVKLLIYHTSRLHSGVPSRFSGSGSGTDYSL
TISNLEQEDIATYFCQQGNTLPYTFGGGTKLEITKAGGGGSGGGGSGGGGSGGGGSEVKLQESGPGLVAP
SQSLSVTCTVSGVSLPDYGVSWIRQPPRKGLEWLGVIWGSETTYYNSALKSRLTIIKDNSKSQVFLKMNS
LQTDDTAIYYCAKHYYYGGSYAMDYWGQGTSVTVSSDPTTTPAPRPPTPAPTIASQPLSLRPEACRPAAG
GAVHTRGLDFACDIYIWAPLAGTCGVLLLSLVITLYCKRGRKKLLYIFKQPFMRPVQTTQEEDGCSCRFP
EEEEGGCELRVKFSRSADAPAYQQGQNQLYNELNLGRREEYDVLDKRRGRDPEMGGKPRRKNPQEGLYNE
LQKDKMAEAYSEIGMKGERRRGKGHDGLYQGLSTATKDTYDALHMQALPPR*

>rapaCasp9-Q8-CAR

MASRILWHEMWHEGLEEASRLYFGERNVKGMFEVLEPLHAMMERGPQTLKETSFNQAYGRDLMEAQEWCR
KYMKSGNVKDLLQAWDLYYHVFRRISKLEYSGGGSLEGVQVETISPGDGRTFPKRGQTCVVHYTGMLEDG
KKFDSSRDRNKPFKFMLGKQEVIRGWEEGVAQMSVGQRAKLTISPDYAYGATGHPGIIPPHATLVFDVEL
LKLESGGGGSGGGGSGGGGSGVDGFGDVGALESLRGNADLAYILSMEPCGHCLIINNVNFCRESGLRTRT
GSNIDCEKLRRRFSSLHFMVEVKGDLTAKKMVLALLELAQQDHGALDCCVVVILSHGCQASHLQFPGAVY
GTDGCPVSVEKIVNIFNGTSCPSLGGKPKLFFIQACGGEQKDHGFEVASTSPEDESPGSNPEPDATPFQE
GLRTFDQLDAISSLPTPSDIFVSYSTFPGFVSWRDPKSGSWYVETLDDIFEQWAHSEDLQSLLLRVANAV
SVKGIYKQMPGCFNFLRKKLFFKTSASRAEGRGSLLTCGDVEENPGPMGLVRRGARAGPRMPRGWTALCL
LSLLPSGFMAELPTQGTFSNVSTNVSPAKPTTTPAPRPPTPAPTIASQPLSLRPEACRPAAGGAVHTRGL

```
DFACDIYIWAPLAGTCGVLLLSLVITLYCNHRNRRRVCKCPRPVVRAEGRGSLLTCGDVEENPGPMETDT
LLLWVLLLWVPGSTGDIQMTQTTSSLSASLGDRVTISCRASQDISKYLNWYQQKPDGTVKLLIYHTSRLH
SGVPSRFSGSGSGTDYSLTISNLEQEDIATYFCQQGNTLPYTFGGGTKLEITKAGGGGSGGGGSGGGGSG
GGGSEVKLQESGPGLVAPSQSLSVTCTVSGVSLPDYGVSWIRQPPRKGLEWLGVIWGSETTYYNSALKSR
LTIIKDNSKSQVFLKMNSLQTDDTAIYYCAKHYYYGGSYAMDYWGQGTSVTVSSDPTTTPAPRPPTPAPT
IASQPLSLRPEACRPAAGGAVHTRGLDFACDIYIWAPLAGTCGVLLLSLVITLYCKRGRKKLLYIFKQPF
MRPVQTTQEEDGCSCRFPEEEEGGCELRVKFSRSADAPAYQQGQNQLYNELNLGRREEYDVLDKRRGRDP
EMGGKPRRKNPQEGLYNELQKDKMAEAYSEIGMKGERRRGKGHDGLYQGLSTATKDTYDALHMQALPPR*
```

Supplementary data-Figure 1

(a)

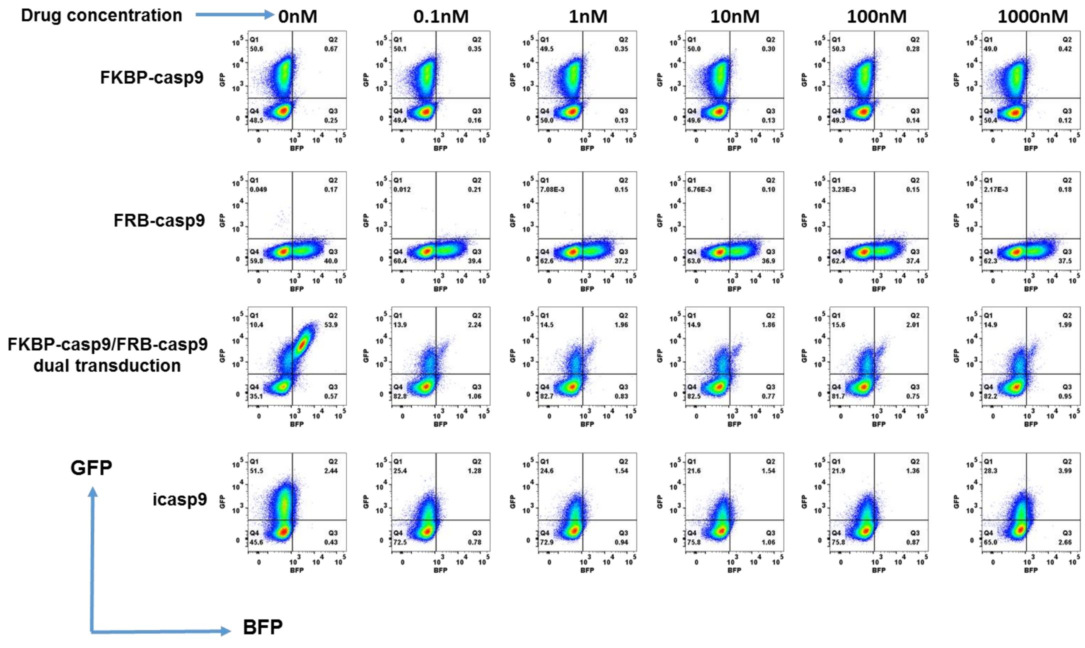

(b)

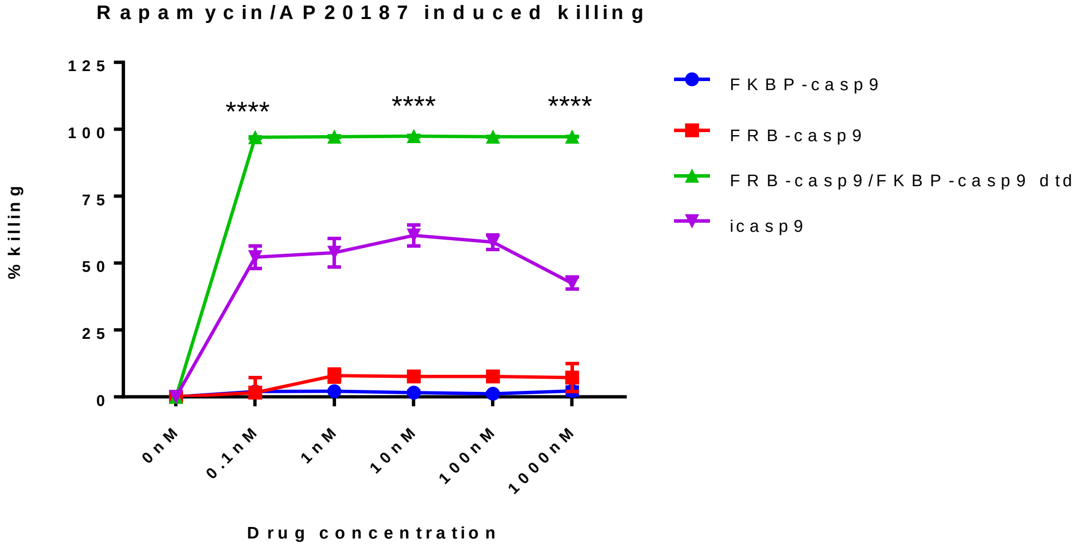

Supplementary data-Figure 2

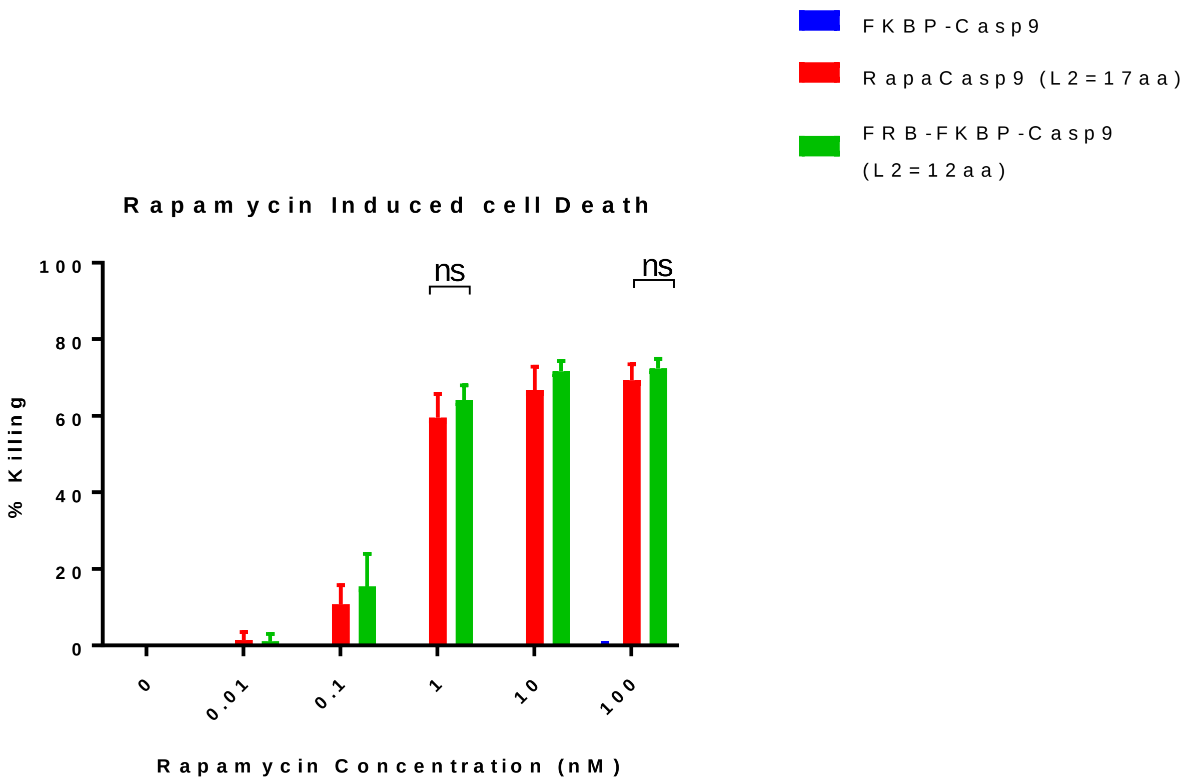

Supplementary data-Figure 3

(a)

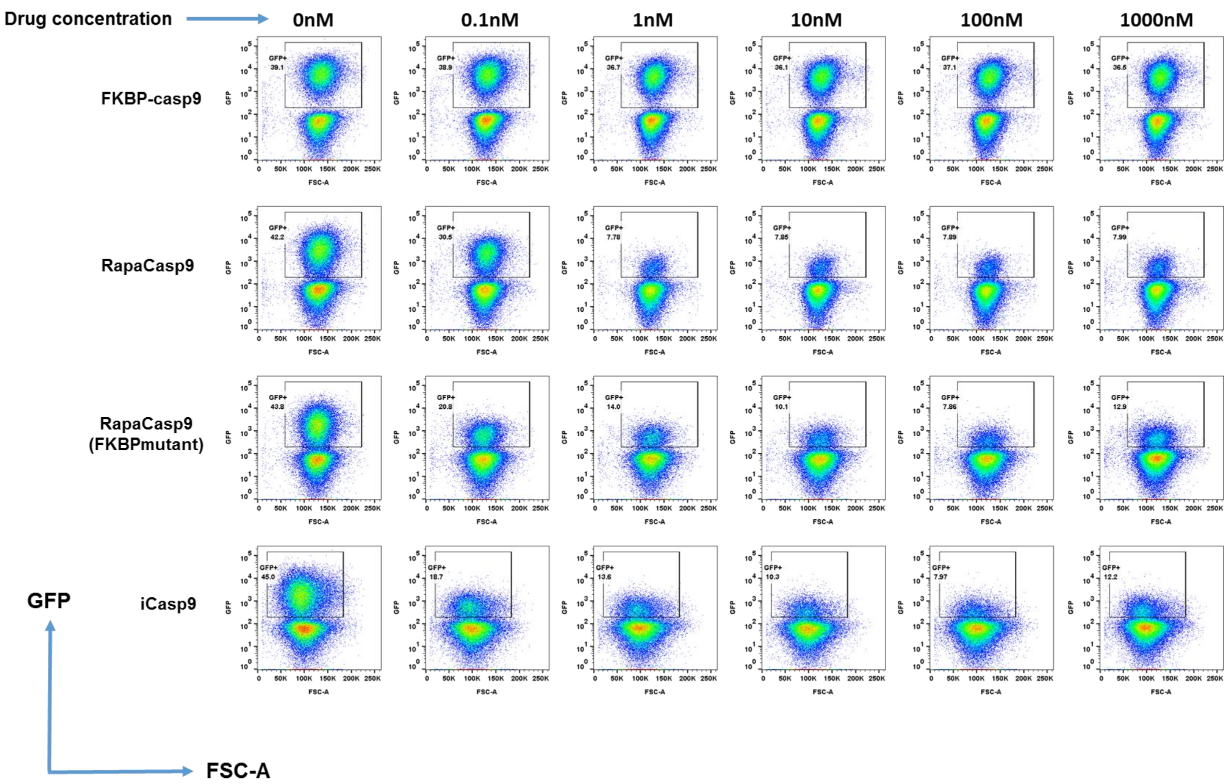

(b)

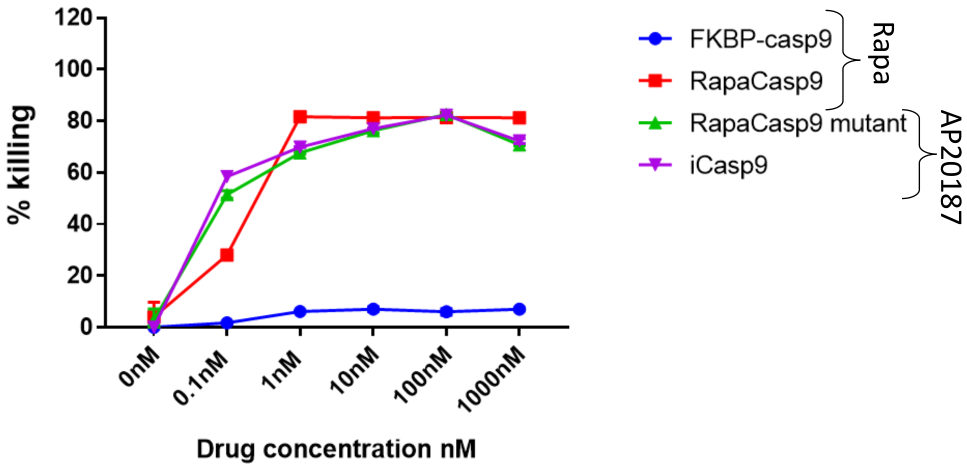

Supplementary data-Figure 4

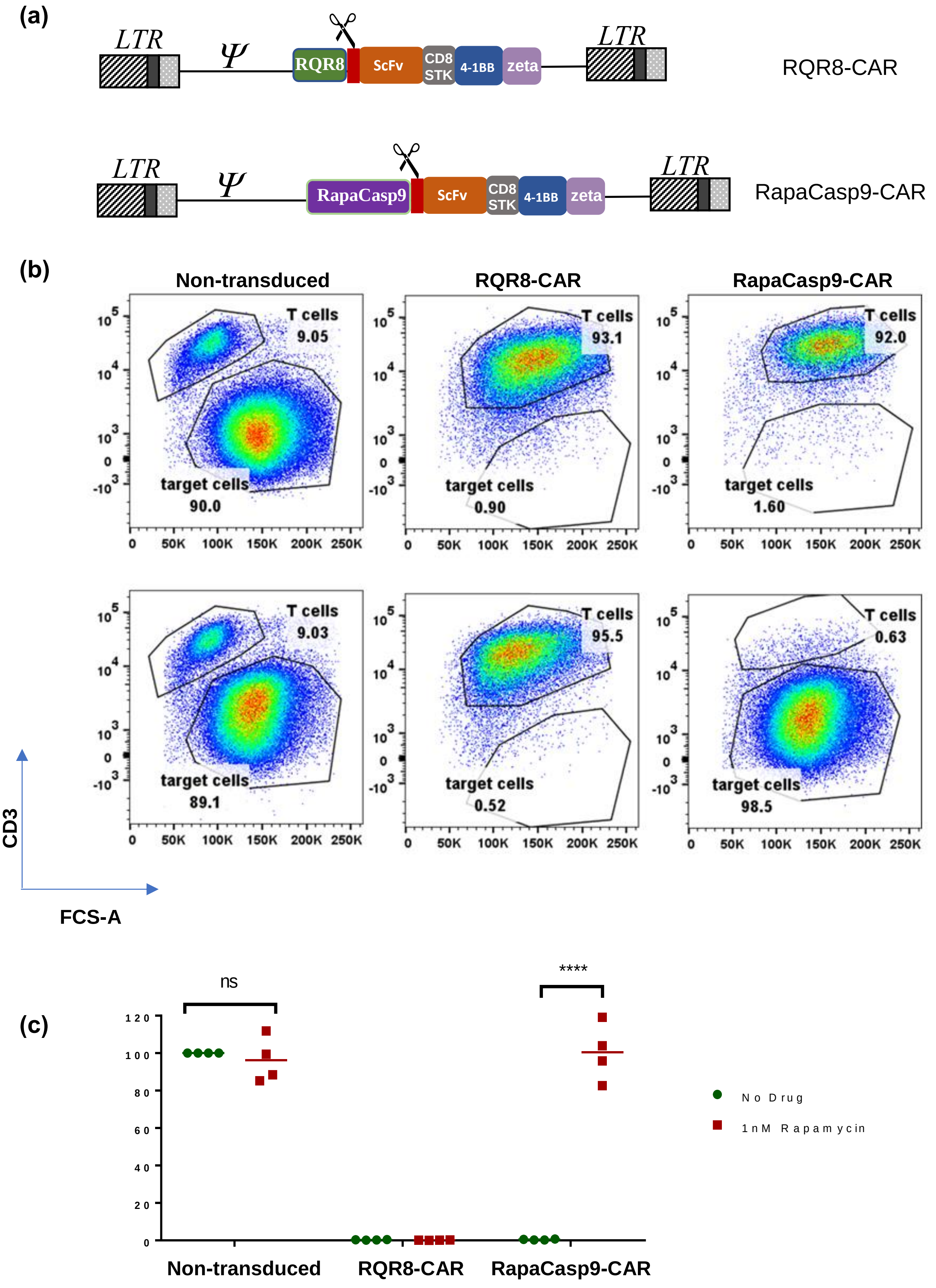

Supplementary data-Figure 5

(a)

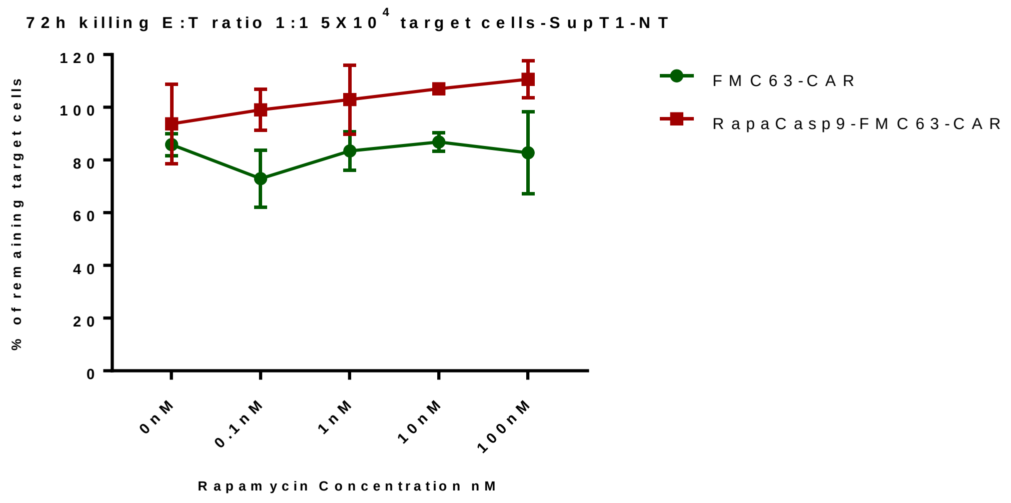

(b)

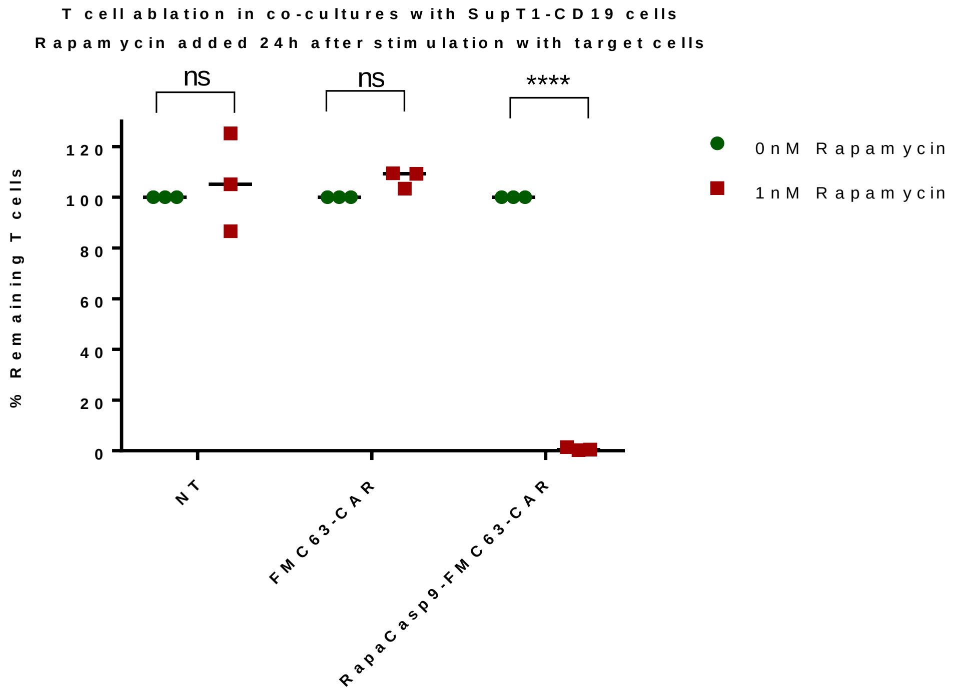

Supplementary data-Figure 6

(a)

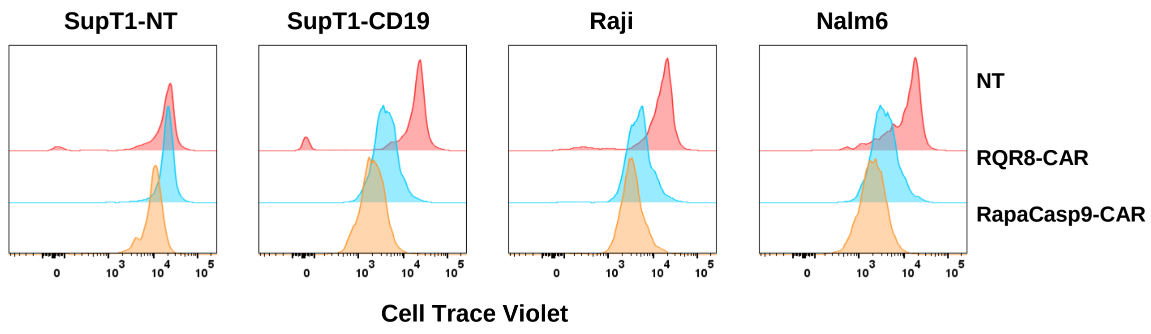

(b)

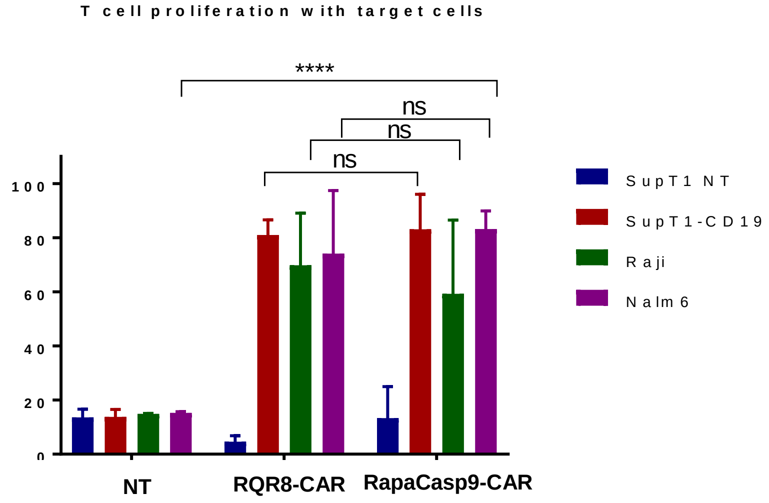

(c)

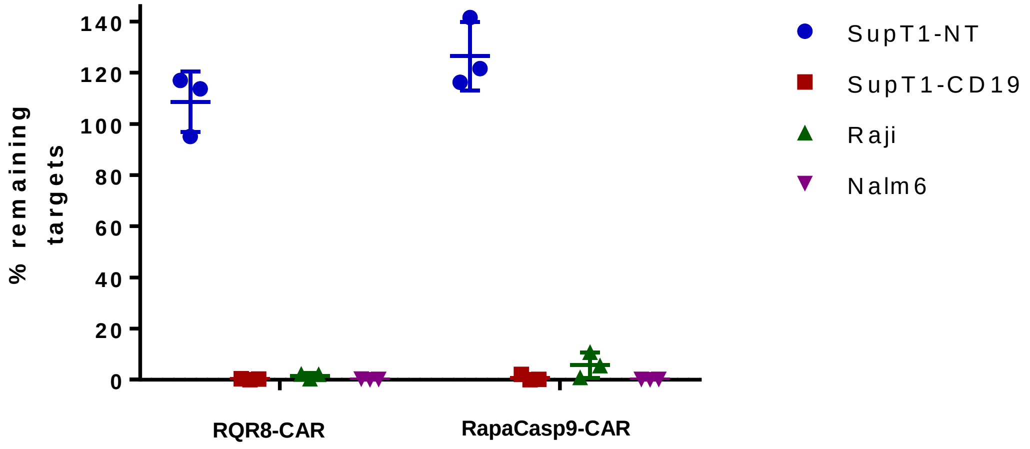

Supplementary data-Figure 6 cont'd

(d)

i)

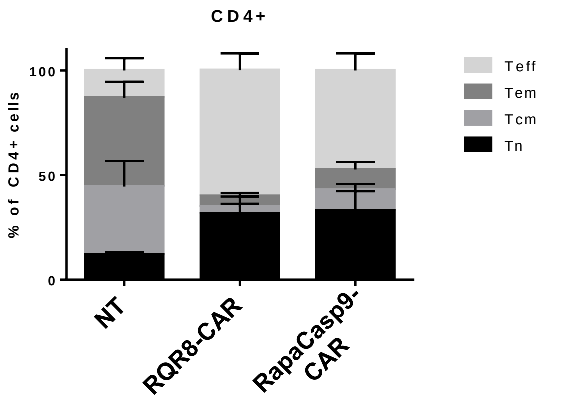

ii)

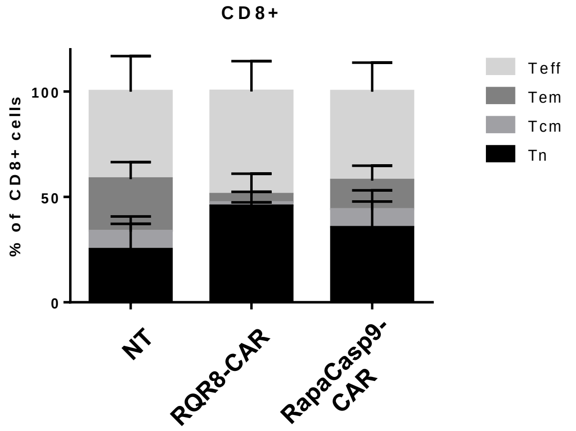

iii)

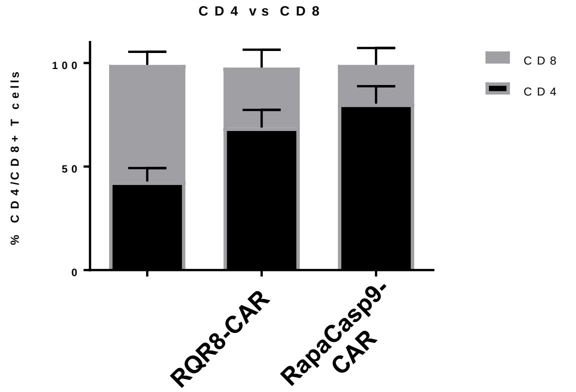

Supplement: Document S1. Amino acid sequences and Figures S1–S6 [file mmc1.pdf]
