# Supplementary material for: A Rapamycin-Activated Caspase 9-Based Suicide Gene
Source: Mol Ther. 2018 Mar 9;26(5):1266–76. doi: 10.1016/j.ymthe.2018.03.001 (PMC5993966; doi:10.1016/j.ymthe.2018.03.001)

Molecular Therapy
Original Article

A Rapamycin-Activated Caspase 9-Based Suicide Gene

Maria Stavrou,[1] Brian Philip,[2] Charlotte Traynor-White,[1] Christopher G. Davis,[1] Shimobi Onuoha,[1] Shaun Cordoba,[1] Simon Thomas,[1] and Martin Pule[1,2]

[1]Autolus Ltd., Forest House, White City, London, UK; [2]Cancer Institute, University College London, London, UK

Engineered T cell therapies show considerable promise in the treatment of refractory malignancies. Given the ability of engineered T cells to engraft and persist for prolonged periods along with unpredicted toxicities, incorporation of a suicide gene to allow selective depletion after administration is desirable. Rapamycin is a safe and widely available immunosuppressive pharmaceutical that acts by heterodimerization of FKBP12 with the FRB fragment of mTOR. The apical caspase caspase 9 is activated by homodimerization through its CARD domain. We developed a rapamycin-induced caspase 9 suicide gene. First, we showed that caspase 9 could be activated by a two-protein format with replacement of the CARD domain with both FRB and FKBP12. We next identified an optimal compact single-protein rapamycin caspase 9 (rapaCasp9) by fusing both FRB and FKBP12 with the catalytic domain of caspase 9. Functionality of rapaCasp9 when co-expressed with a CD19 CAR was demonstrated *in vitro* and *in vivo*.

INTRODUCTION

Adoptive transfer of antigen-specific T cells is finding increasing application as a cancer therapy. Adoptively transferred T cells can have a native specificity, such as *ex vivo*-expanded tumor-infiltrating lymphocytes or donor lymphocyte infusions following hematopoietic stem cell transplantation (HSCT). T cells can also be genetically engineered with a chimeric antigen receptor (CAR) or transgenic T cell receptor (TCR). The complexity and potency of engineered T cells is increasing, and severe unexpected toxicity occurs.[1–4] Because engineered T cells engraft and persist indefinitely, the ability to control T cells after administration is a desirable feature of an experimental T cell therapeutic agent.

Suicide genes are genetically encoded elements that allow selective destruction of expressing cells in the face of unacceptable toxicity by administration of an activating pharmaceutical agent. Several suicide genes have been described. One family of suicide genes, such as RQR8[5] and huEGFRt,[6] are surface proteins recognized by therapeutic monoclonal antibodies (mAbs). T cells can be depleted by administration of the cognate therapeutic mAbs. A possible limitation of mAb-mediated suicide genes is the requirement for the therapeutic mAb at sufficient concentrations to be active, which may not be achieved in all tissue distributions.

Other suicide genes have been described that are activated by a small molecule. Two of these have been tested in clinical studies: herpes simplex virus thymidine kinase (HSV-TK)[7] and inducible caspase 9 (iCasp9).[8] Expression of HSV-TK in T cells confers susceptibility to ganciclovir. HSV-TK is a highly effective suicide gene strategy, but immunogenicity[9] limits application to clinical settings of profound immunosuppression, such as haploidentical HSCT. Further, it precludes the use of ganciclovir for the treatment of cytomegalovirus infection.

iCasp9 is a fusion of a mutated FKBP12 with the catalytic domain of caspase 9.[10] The FKBP12 is mutated to allow docking of a small molecular chemical inducer of dimerization (CID, AP1903/AP20187) that cannot bind wild-type (WT) FKBP12 and is, hence, otherwise pharmacologically inert.[11] Because iCasp9 is a fusion of self-proteins, it is unlikely to be immunogenic. Like HSV-TK, iCasp9 utility has been proven in a clinical study of haploidentical HSC transplantation.[12]

A practical limitation of iCasp9 is the requirement for an experimental small molecule that is not a licensed pharmaceutical agent. An alternative small-molecule dimerizer is rapamycin (and its semi-synthetic analogs).[13] Rapamycin is not pharmacologically inert, being an immunosuppressive drug. However, in the context of a very short dosing schedule to activate a suicide gene and deplete T cells, pharmacological activity would be minimal and may even help deplete the transgenic T cells. A rapamycin-induced suicide gene would be a convenient component for engineered T cell therapy.

Although iCasp9-activating CIDs homodimerize FKBP12, rapamycin heterodimerizes the FKBP12-rapamycin binding domain (FRB) fragment of mammalian target of rapamycin (mTOR) with FKBP12.[14] Caspase 9 activation requires homodimerization[15] and perhaps higher-order oligomerization.[16] We show that, using a two-component system, it is possible to effectively activate caspase 9 with

Received 17 September 2017; accepted 3 March 2018;
https://doi.org/10.1016/j.ymthe.2018.03.001.

Correspondence: Martin Pule, Cancer Institute, University College London, 72 Huntley Street, London WC1E 6BT, UK.
E-mail: m.pule@ucl.ac.uk

This is an open access article under the CC BY license (http://creativecommons.org/licenses/by/4.0/).

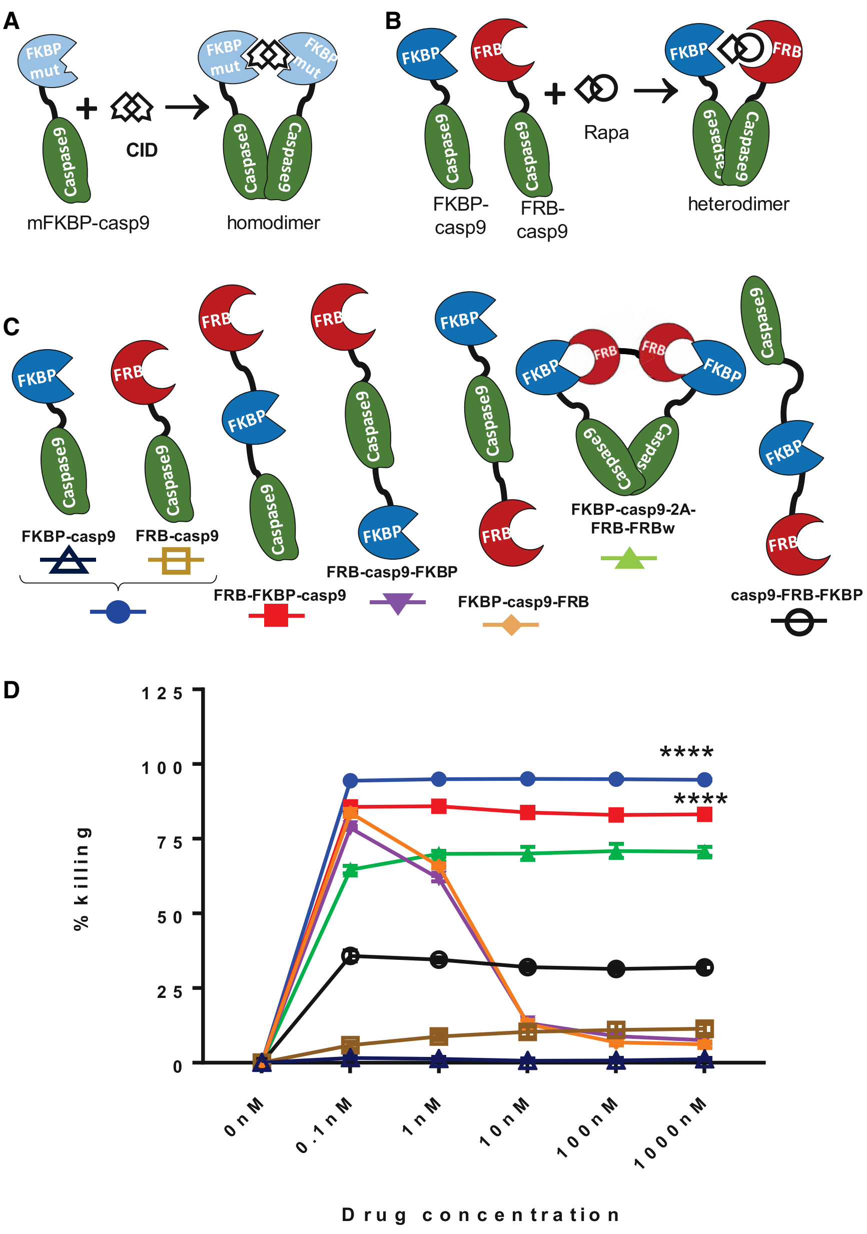

Figure 1. Function of Rapamycin-Induced Suicide Constructs

(A) Schematic representation of iCasp9 and a rapamycin-based suicide gene. In iCasp9, a mutant FKBP domain fused to the caspase 9 catalytic domain allows homodimerization upon administration of a chemical inducer of dimerization, CID (AP20187/AP1903). (B) Rapamycin-induced activation requires hetero-dimerization of the FRB-caspase 9 fusion and the FKBP-caspase 9 fusion. (C) Compact variants of rapamycin-caspase 9 suicide genes tested (N terminus, top, through to C terminus, bottom). FKBP-Casp9/FRB-Casp9 alone or together are controls. With FKBP-Casp9-2A-FRB-FRBw, FKBP-Casp9 was co-expressed with two linked FRBs (the second being codon-wobbled) using a foot-and-mouth 2A peptide co-expression. Suicide genes were co-expressed with EGFP using an IRES sequence (or eBFP2 for FRB-Casp9). (D) Jurkat cells transduced with the above constructs were treated with increasing concentrations of rapamycin (0.1 to 1,000 nM). Rapamycin-induced cell death was assessed 24 hr later, after Annexin V/7-AAD staining, by flow cytometry. The percentage of killing reported was the percentage of transduced live cells relative to the untreated control for each condition. Results are from 4 independent experiments (n = 4). Statistical analysis was performed using repeated measures two-way ANOVA with Dunnett's post-test for multiple comparisons. **** indicates the significantly higher cell killing observed in the corresponding populations at 0.1, 1, 10, 100, and 1,000 nM rapamycin compared with the FKBP-Casp9 negative control. ****p = 0.0001. Error bands correspond to the mean with SD of four independent experiments.

rapamycin. From this starting point, we optimized a single-component rapamycin-activated caspase 9 (rapaCasp9). This suicide gene has equivalent function to iCasp9 but can be activated with an off-the-shelf pharmaceutical agent.

RESULTS

Co-expressed FRB-Caspase 9/FKBP12-Caspase 9 Can Be Activated by Rapamycin

We first sought to demonstrate whether caspase 9 (Figure 1A) could be engineered to be activated by rapamycin in a comparable manner to iCasp9 activation, using the simplest possible construct: the FRB domain of mTOR fused to the catalytic domain of caspase 9 (FRB-Casp9) co-expressed with FKBP12 fused to the caspase 9 catalytic domain (FKBP-Casp9) (Figure 1B). Jurkat cells were transduced with FRB-Casp9, FKBP-Casp9 or both. Co-expression of EGFP and enhanced blue fluorescent protein 2 (eBFP2) marker genes allowed determination of transduction efficiency. As a control, Jurkat cells were also transduced with iCasp9 co-expressed with EGFP. Jurkat cells were treated with a range of 0.1 nM to 1,000 nM of either rapamycin or AP20187. Live cells expressing the suicide constructs, defined as those that were negative for both 7-amino-actinomycin D (7-AAD) and Annexin V staining and positive for EGFP/eBFP2 were enumerated by flow cytometry, and the percentage of cell death was calculated by normalizing these values to the respective untreated control (Figures S1A and S1B). FRB-Casp9/FKBP-Casp9 resulted in highly efficient induction of apoptosis from 0.1 nM rapamycin, whereas, as expected, FRB-Casp9 or FKBP-Casp9 alone did not induce apoptosis. Notably, FRB-Casp9/FKBP-Casp9 Jurkat cells demonstrated a flat dose-response curve with no attenuation at the highest levels of rapamycin tested.

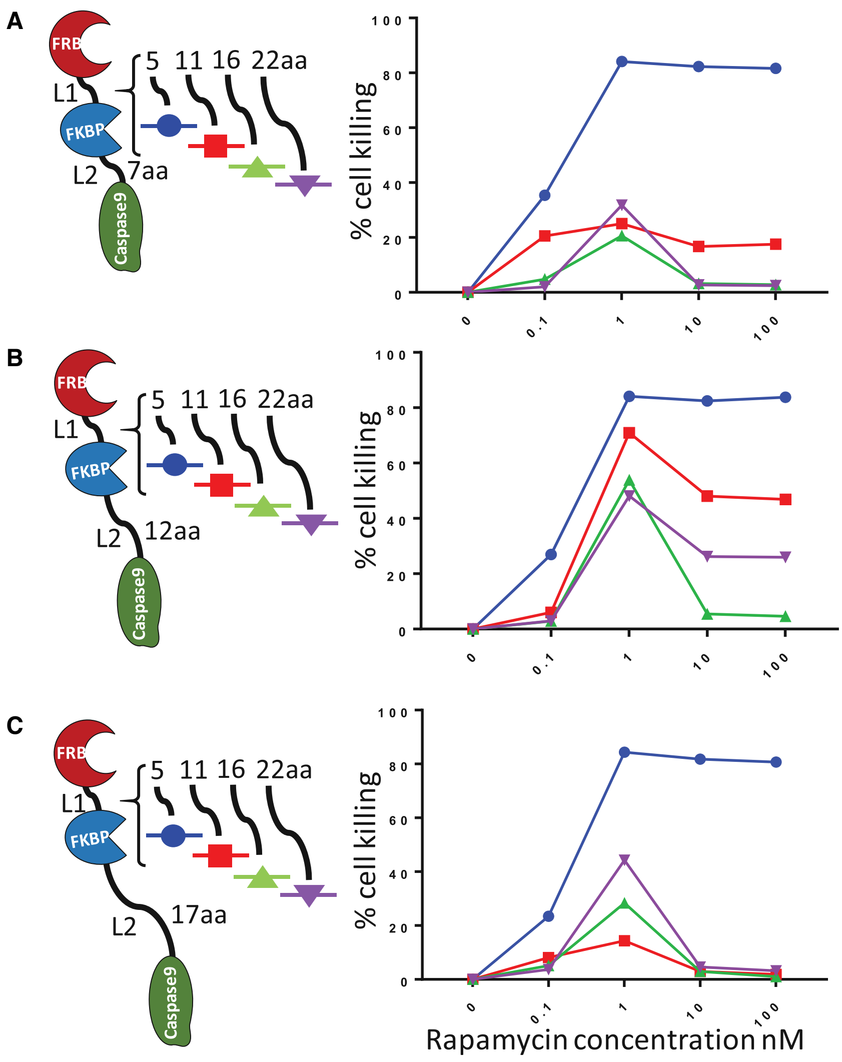

Figure 2. Exploration of FRB-FKBP12-Casp9 Structure/Function

Variants of FRB-FKBP-Casp9 with different-length Ser-Gly linkers between FRB-FKBP (linker 1 [L1]) and FKBP-Casp9 (linker 2 [L2]) were tested. (A) FRB-FKBP-Casp9 variants with a 7-aa L2 combined with L1 of either 5 aa, 11 aa, 16 aa, and 22 aa were tested for sensitivity to rapamycin. (B) FRB-FKBP-Casp9 with a 12-aa L2 combined with different L1 lengths was tested. (C) FRB-FKBP-Casp9 variants with a 17-aa L2 combined with different L1 lengths was tested. Jurkat cells were transduced with the indicated constructs and treated with increasing concentrations of rapamycin (0.1 to 100 nM). Cells were incubated for 24 hr, and rapamycin-induced cell killing was assessed by flow cytometry. Cell killing was calculated as the percentage of transduced live cells of the respective untreated control. Results are from 2 independent experiments (n = 2).

Compact Rapamycin-Induced Caspase9 Suicide Systems

The above data demonstrate the feasibility of the use of a rapamycin-activated caspase 9. However, this format is not practical because it requires co-expression of two long reading frames with a high degree of homology, posing an increased risk of recombination. We therefore sought to construct a more compact version that could contain all the required elements in a single retroviral cassette. The variants tested are illustrated in Figure 1C: several constructs contained FKBP12, FRB, and the catalytic domain of caspase 9 as a single fusion protein in different orientations. Alternatively, an adaptor protein formed of two copies of FRB was co-expressed with FKBP-Casp9.

Jurkat cells were transduced with the different rapamycin-induced suicide constructs. Jurkat cells singly or doubly transduced with FKBP-Casp9/FRB-Casp9 were used as controls. The cells were exposed to increasing concentrations of rapamycin ranging from 0.1 nM to 1,000 nM for 24 hr. Cell death is shown in Figure 1D. All new constructs induced apoptosis at varying degrees upon exposure to rapamycin; however, the compact construct displaying the greatest level of efficacy was FRB-FKBP-Casp9. This construct has the rapamycin binding domains positioned adjacent to each other at the N terminus of the casp9 catalytic domain.

Positioning the rapamycin binding domains on the C terminus of the casp9 catalytic domain (casp9-FRB-FKBP) diminished activity considerably. Notably, the dose-response curve was flat with FRB-FKBP constructs. However, two constructs in which FRB/FKBP12 flanked the caspase 9 catalytic domain showed a bimodal response curve. The bicistronic constructs with an adaptor element resulted in reduced overall activity.

Further Study of Structure-Function of the FRB-FKBP-Casp9 Construct

Having established that FRB-FKBP-Casp9 was the optimal configuration, we set about exploring and perhaps optimizing this configuration. The three domains are connected via two Ser-Gly linkers that are referred to as L1, connecting FRB to FKBP, and L2, connecting FKBP to casp9, respectively (Figure 2). These linkers must allow sufficient flexibility for a staggered interaction but must not allow intra-molecular ligation of rapamycin. We therefore tested several variants with different combinations of L1 and L2 linker lengths. Increasing L1 to more than 5 amino acids (aa) leads to diminished response and a bimodal dose-response curve independent of L2 at all tested concentrations of rapamycin. A possible explanation for the effects of increasing L1 is an increasing propensity for intra-molecular binding

of rapamycin to both FRB and FKBP12. The L2 linker length of 12 amino acids improved function with a long L1 but had no effect on function with a short L1. To see whether a benefit of a longer L2 with a 5-aa L1 could be unmasked in primary T cells, constructs with L1 of either 12 or 17 aa were compared (Figure S2). No difference was observed. In this experiment, the dose-response curve was extended down to 0.01 nM.

One possible advantage of iCasp9 is the absence of non-productive interactions between the iCasp9 molecule and endogenous FKBP12. To test the consequence of possibly non-productive FKBP12 and mTOR interactions, we generated a version of FRB-FKBP12-Casp9 where the FKBP12 had the F36V mutation to accommodate AP20187. Activation with AP20187 was similar to that with rapamycin (Figure S3), suggesting that non-productive interactions do not affect rapamycin-induced caspase 9 performance.

RapaCasp9 Activity in Primary T Cells: T Cell Death and Effect on Function

Subsequent experiments used FRB-L1:5aa-FKBP-L2:17aa-Casp9 (henceforth referred to as rapaCasp9). Because iCasp9 function in primary T cells depends on transgene expression,[9] we tested both suicide genes in T cells either sorted for high transgene expression or unsorted. Primary T cells were retrovirally transduced with either the rapaCasp9 or the iCasp9 constructs that co-expressed EGFP. Transduced cells were sorted into GFP^{high}-expressing cells by flow sorting or left unsorted.

T cells from both the high-expressing and the unsorted populations were exposed to increasing concentrations of rapamycin or AP20187 (0.1 nM to 100 nM) for 24 hr. Cell death was assessed by flow cytometry. Representative flow cytometry plots of live gated unsorted cells or GFP^{high} sorted cells treated with increasing concentrations of the appropriate drug from a single experiment are shown in Figure 3A for iCasp9 construct and Figure 3B for rapaCasp9. The same experiment was repeated using 6 different peripheral blood mononuclear cell (PBMC) donors, and cumulative data are shown in Figures 3C and 3D for iCasp9 and rapaCasp9, respectively. Our data show that rapaCasp9 is more efficient within the GFP^{high} population. As observed before, the cells that escape killing are those expressing low levels of the transgene. iCasp9 activity appears similar, but survival of low-expressing cells appears less pronounced. We conclude that T cell products would need to be sorted for high levels of rapaCasp9 to ensure sensitivity to rapamycin of the entire infused population.

We next tested the performance of rapaCasp9 co-expressed with a CAR in flow-sorted primary human T cells. RapaCasp9 was co-expressed with an FMC63-based CD19 CAR[17] in a single retroviral cassette using a foot-and-mouth disease 2A-like peptide (a construct termed "rapaCasp9-CAR")[18] (Figure S4A). A control construct was used that had the same CAR co-expressed with RQR8[5] instead of rapaCasp9 ("RQR8-CAR"). Transduction efficiency was determined by staining with soluble CD19-Fc fusion (sCD19). T cells transduced with either construct were sorted for CAR^{high} expression and co-cultured at a 1:1 ratio with SupT1-WT (negative for CD19) or SupT1-CD19 cells. Co-cultures were set up in the absence or presence of 1 nM of rapamycin. T cell/target cell survival was determined by flow cytometry. Representative plots and cumulative data from 4 different donors are shown in Figures S4B and S4C. These data suggest that T cells expressing rapaCasp9 can exert their full killing potential upon encountering their CD19 target antigen in the absence of rapamycin while retaining their full sensitivity to rapamycin-induced cell ablation.

Performance of T Cells Transduced with rapaCasp9 Co-expressing a Clinically Useable Selection Marker

To test the clinical utility of rapaCasp9, using an additional 2A peptide, we incorporated a clinically useable selection marker, Q8,[5] into the above rapaCasp9-CAR construct to generate rapaCasp9-Q8-CAR. Q8 consists of the minimal epitope of human CD34 recognized by the mAb QBEND/10 attached to a CD8 stalk and transmembrane (TM) domain. This allows selection by Miltenyi Biotec CD34 cliniMACS beads (to which QBEND/10 is conjugated). The RQR8-CAR construct described above was used as a control and also allows QBEND/10 sorting (Figure 4A).

Peripheral blood T cells transduced with either construct were stained with sCD19 and QBEND/10. Flow cytometric analysis demonstrated co-expression of CAR and the marker gene (Figure 4B). Subsequent sorting of the T cells with CD34 Miltenyi microbeads allowed the recovery of pure $CAR^{+}QBend10^{+}$ cell populations (over 97% double-positive) with high mean fluorescence intensity (MFI) (Figure 4B). Comparison of unsorted with bead-sorted cells from 3 individual donors revealed a significant increase in the percentage of the double $CAR^{+}QBend10^{+}$ population, which was very close to 100% for both the RQR8-FMC63CAR and rapaCasp9-Q8-FMC63CAR constructs (Figure 4C).

The sensitivity of bead-sorted CAR T cells to rapamycin was investigated by treating the sorted populations with increasing concentrations of rapamycin. The representative flow cytometry plots in Figure 4D show the efficient ablation of the sorted rapaCasp9-Q8-CAR T cells with concentrations as low as 0.1 nM rapamycin. The percentage of T cells killed at different concentrations of rapamycin in 3 donors is presented in the graph in Figure 4E. Almost complete ablation of the T cells expressing the rapaCasp9 is observed starting from 0.1 nM rapamycin with a flat dose-response curve (from 0.1 to 100 nM). Next, the ability of these untreated and rapamycin-treated CAR T cell populations to lyse Raji cells was tested. In co-cultures, efficient killing of Raji cells by rapaCasp9-Q8-CAR T cells is observed in the absence of rapamycin but is considerably attenuated even after exposure to 0.1 nM (Figure 4F). Killing of CD19 negative targets is shown in Figure S5A. The response of bead-sorted rapaCasp9-Q8-CAR T cells exposed to rapamycin *after* stimulation was also tested (Figure S5B).

Finally, the phenotype and ability of rapaCasp9-Q8-CAR T cells to proliferate and lyse different $CD19^{+}$ cell lines were compared with

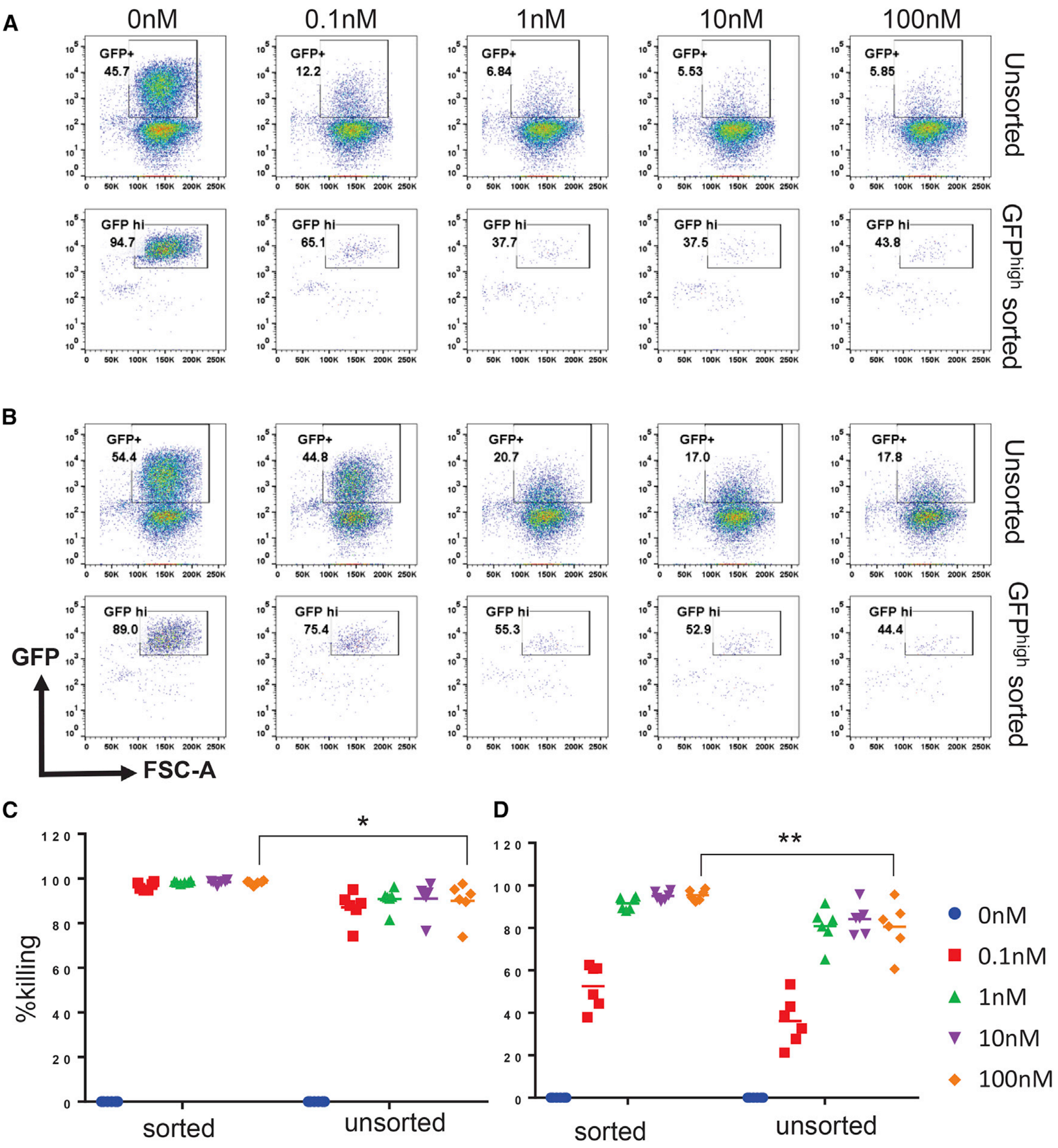

Figure 3. Function of rapaCasp9 in Primary Human T Cells

Peripheral blood T cells were transduced with either iCasp9 or rapaCasp9 (EGFP was co-expressed using an IRES). Unsorted transduced T cells or T cells sorted for high GFP expression were tested for responsiveness to AP20187 or rapamycin, respectively. T cells were treated with the drugs at concentrations ranging from 0.1 to 100 nM. After 24 hr incubation, cell death was determined using Annexin V/7-AAD staining by flow cytometry. (A) Representative plots for iCasp9 with EGFP shown against the forward scatter area (FSC-A). (B) Representative plots for rapaCasp9 with EGFP also shown against the FSC-A. (C) Percentage of iCasp-transduced T cells killed by AP20187 over untreated controls within the sorted EGFP-high and unsorted populations at different concentrations of the drug. (D) Percentage of rapaCasp9-transduced T cells killed by rapamycin over untreated controls within the EGFP-high and unsorted populations at different concentrations of the drug. Lines indicate the mean value of each condition for 6 separate donors. Statistical analysis was carried out using repeated measures two-way ANOVA with Sidak's post-test for multiple comparisons. *$p < 0.02$, **$p < 0.005$.

that of RQR8-CAR T cells. RapaCasp9-expressing CAR T cells showed identical proliferation in response to SupT1 cells, SupT1.CD19 cells, Raji cells, and NALM6 cells as control CAR T cells (Figures S6A and S6B). Similarly, there was no difference between the rapaCasp9-CAR and the control CAR-expressing T cells in their ability to kill target cells (SupT1.CD19, Raji, and Nalm6) (Figure S6C). Phenotypic analysis showed no statistically significant difference (Figure S6D).

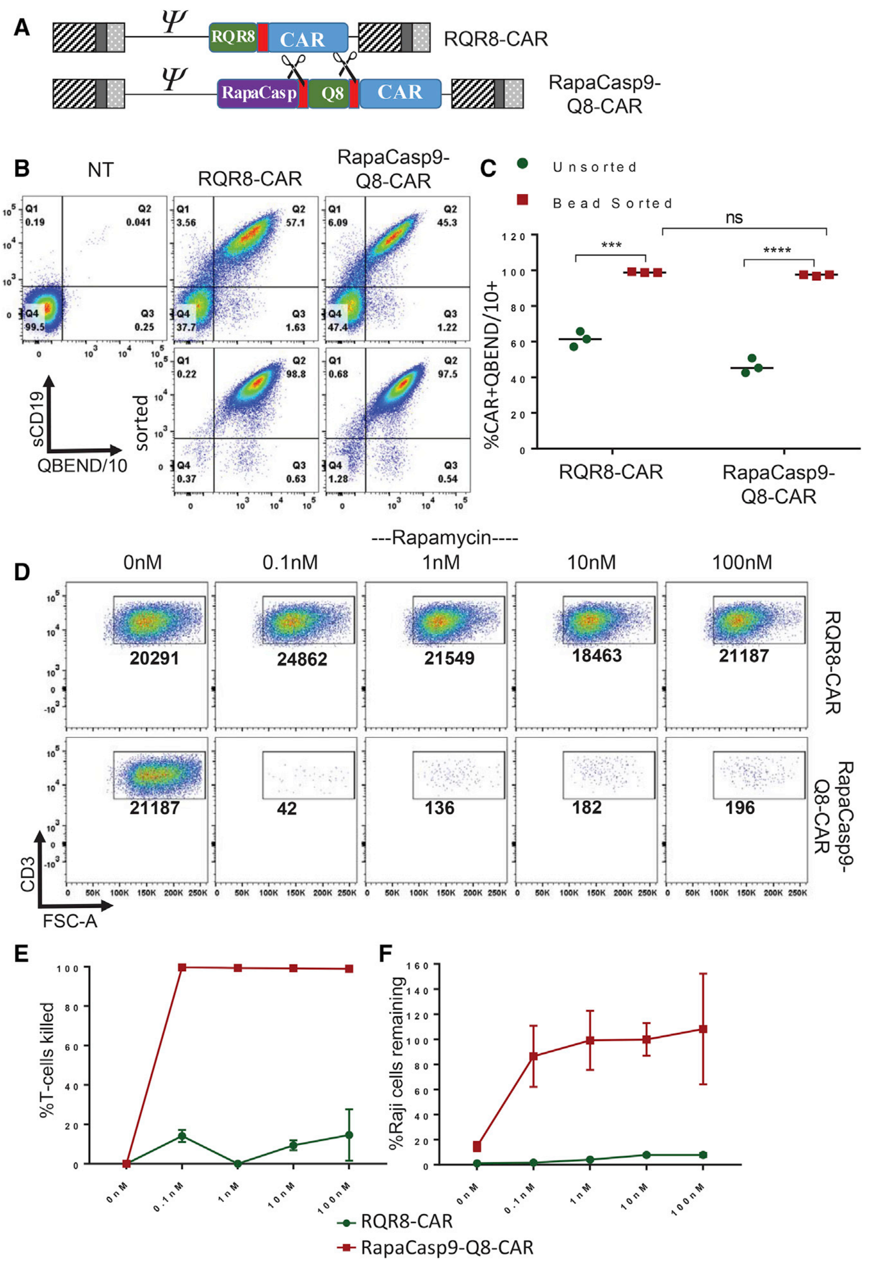

Figure 4. Testing rapaCasp9 as Part of a CD19-CAR Construct

(A) Schematic representation of RQR8-CAR and rapaCasp9-Q8-CAR. T cells transduced with these constructs were stained with sCD19 and with the QBEND/10 mAb to demonstrate co-expression of CAR and marker genes. T cells were then sorted using Miltenyi CD34 beads (which utilize the QBEND/10 mAb). (B) Representative flow cytometry plots of non-transduced, sorted, and unsorted T cells transduced with either construct. The percentage of cells positive for CAR and marker is shown in the plots. (C) The percentage of CAR$^+$CD34$^-$marker$^+$ cells for both unsorted and magnetic bead-sorted cells is shown for 3 individual PBMC donors. Statistical analysis was carried out using repeated measures two-way ANOVA with Sidak's post-test for multiple comparisons. ****p < 0.0001, ***p = 0.0002. These sorted populations from both RQR8-CAR- and rapaCasp9-Q8-CAR-transduced cells were treated with increasing concentrations of rapamycin (0–100 nM). Surviving T cells were determined by flow cytometry after staining with 7-AAD. Normalized numbers of live cells are shown. Representative flow cytometry plots from one donor after staining with 7-AAD and CD3 are shown in (D). The percentage of T cell killing in 2 individual donors is shown in the graph in (E). Raji cells were co-cultured with either RQR8-CAR or rapaCasp9-CAR T-cells at an E:T ratio 1:1. Co-cultures were set up in the absence of rapamycin or at increasing concentrations of rapamycin (0.1-100nM). The percentage of remaining Raji cells is shown in the graph in (F). Error bars in (E) and (F) correspond to mean with SD of the results from two donors.

Rapamycin Induces *In Vivo* Ablation of T Cells Expressing rapaCasp9-FMC63-CAR

To evaluate the function of rapaCasp9 in transduced T cells *in vivo*, we used an NOD.Cg-*Prkdc^{scid} Il2rg^{tm1Wjl}*/SzJ (NSG) mouse-human xenograft model for adoptive immunotherapy. Mice were injected with 2.5 × 10^5 tumor cells (Raji cells) for the provision of hCD19 stimuli, and 4 days later, they received an intravenous (i.v.) injection of 2.5 × 10^5 T cells expressing firefly luciferase and either RQR8-CAR or rapaCasp9-CAR. Cells were sorted to CAR^{high}-expressing cells prior to injection into the animals. Bioluminescent imaging (BLI) 3 days after T cell infusion confirmed the T cell engraftment in mice. On the same day, mice were either treated with a single dose of rapamycin (5 mg/kg) or were left untreated. BLI carried out 3 days after drug administration allowed the evaluation of cell death induced by rapamycin.

BLI showed almost complete absence of signal in the mice that received T cells expressing rapaCasp9-CAR after they were treated with Rapamycin (day 3 after treatment). In contrast, in mice injected with T cells expressing the same construct that did not receive rapamycin, a considerable expansion of T cells was observed, shown by increased radiance (Figures 5B and 5C). In mice that received RQR8-CAR-transduced T cells, T cells expanded both in mice treated with rapamycin and the ones not receiving the drug, as shown by the increase in radiance compared with the first BLI measurement. However, we observed that T cells show poorer expansion in mice receiving rapamycin, which is expected based on its immunosuppressive effects (Figures 5A and 5C).

We next sought to compare the function of rapaCasp9 with iCasp9. T cells were transduced with either rapaCasp9-CAR or iCasp9-CAR

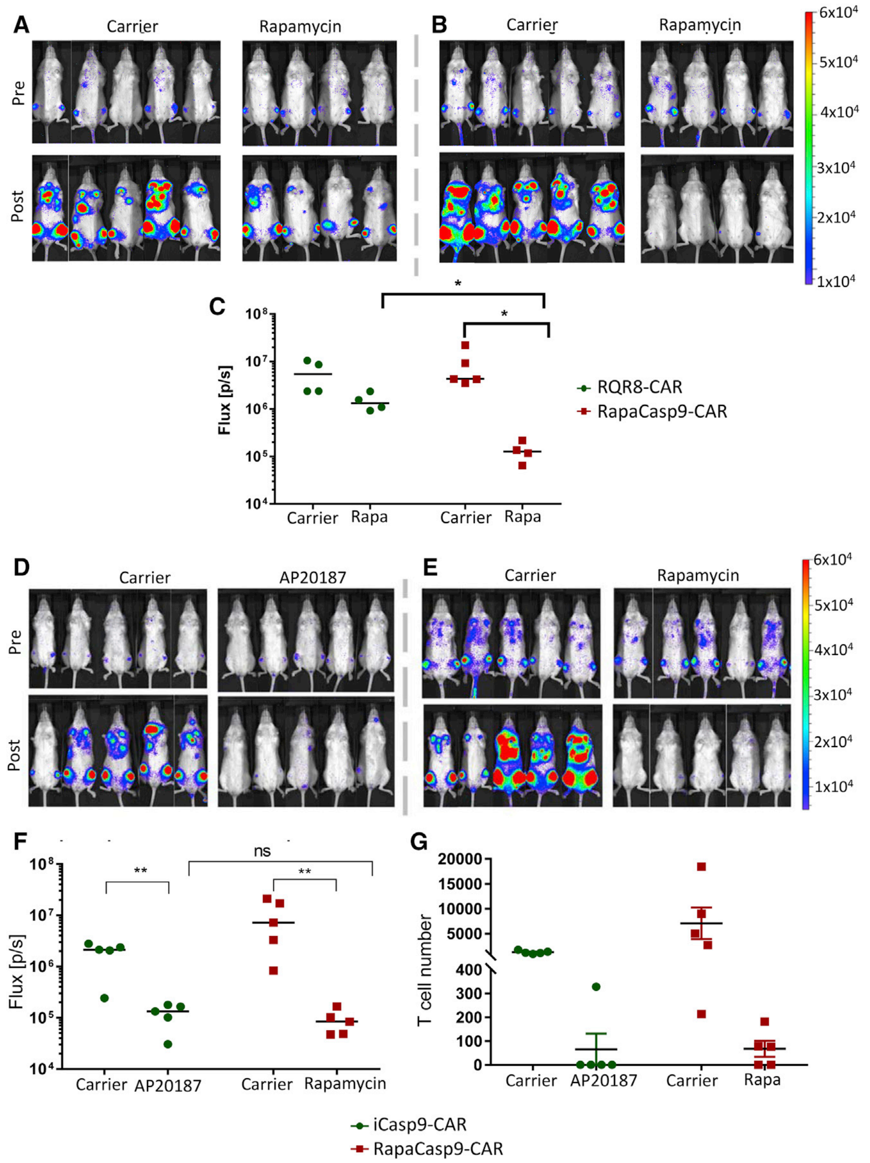

Figure 5. In Vivo Evaluation of the rapaCasp9 Construct

Mice were injected with 2.5×10^5 tumor cells (Raji cells) for the provision of hCD19 stimuli. Four days later, they received an intravenous injection of 2.5×10^5 T cells expressing firefly luciferase and either CAR or rapaCasp9-CAR sorted for CAR^{high} expression. Engraftment of the injected T cells was assessed by BLI 3 days after i.v. injection. On the same day, mice injected with T cells expressing each of the 2 CARs were separated into 2 groups, of which one was injected with 5 mg/kg rapamycin and the other with carrier alone. BLI was performed 3 days later (day 6 after CAR T cells). (A) BLI of mice receiving CAR only-expressing T cells before rapamycin/carrier (i.e., on day 3 after CAR T cell infusion, labeled "pre") and after rapamycin/carrier (i.e., day 6 after CAR T cell infusion, labeled "post"). (C) The total radiance detected in mice after rapamycin injection (day 6 after CAR T cell infusion) is shown in the graph. (D) A similar experimental setup as the one described above was used for *in vivo* comparison of the rapaCasp9-CAR with the iCasp9-CAR construct. Sorted T cells transduced with either construct were injected with 4×10^5 cells per mouse in mice pre-injected with Raji tumor cells. BLI was carried out 3 days after T cell injection. On the same day, mice injected with iCasp9-CAR-expressing T cells were split into 2 groups receiving either carrier or 50 μg of AP20187 (CID). Similarly, the mice injected with rapaCasp9-CAR T cells were split into 2 groups receiving either carrier or 100 μg rapamycin. BLI was carried out 3 days later to assess T cell persistence. (D) BLI of mice treated with iCasp9-CAR T cells before and after carrier/AP20187. (E) BLI of mice treated with rapaCasp9-CAR T cells before and after carrier/rapamycin. (F) The total radiance detected in mice after carrier/AP20187/rapamycin injection. (G) Absolute T cell number in the BM. This was calculated after harvesting the BM from one leg from each mouse and carrying out flow cytometry for the detection of T cells in the sample. Statistical analysis was performed using two-tailed, non-parametric, unpaired t test (Mann-Whitney). Error bands correspond to the mean with SEM of the measures from five mice. *$p < 0.03$, **$p < 0.01$.

along with firefly luciferase and administered to Raji cell-bearing mice in an identical manner as in the experiment described above. Mice receiving rapaCasp9-expressing T cells were either treated with a single dose of rapamycin or carrier alone; mice receiving iCasp9-expressing T cells were treated with AP20187 or carrier alone. BLI showed almost complete absence of signal in all mice treated with either rapamycin or AP20187 (Figures 5D–5F). Bone marrow aspirate was also studied by flow cytometry for surviving CAR T cells. No difference in T cell depletion between rapaCasp9 and iCasp9 was observed (Figure 5G).

DISCUSSION

Adoptively transferred T cells can cause toxicity. For instance, donor lymphocytes in the setting of HSCT can cause graft versus host disease. T cells with engineered specificities can result in toxicities that are sometimes unpredictable. Further, CAR T cells directed against CAIX and ERBB2 as well as TCRs directed against carcinoembryonic antigen (CEA) resulted in on-target off-tumor toxicity.[2,19,20] In addition, non-specific TCR recognition has caused fatal cardiac toxicity.[3] Further still, non-specific effects have caused severe and fatal toxicity, such as neurotoxicity, after CD19 CAR therapy.[21] Notably, pre-clinical testing has not predicted many of these toxicities. Suicide genes allow mitigation of unexpected toxicities and can increase the safety and, hence, speed of clinical development of engineered T cells.

Several different suicide gene approaches have been described.[22] Arguably the best suicide gene described for T cell therapy at present is iCasp9.[10] This suicide gene has a short coding sequence; it is a

fusion of two self-proteins, so it is unlikely to be immunogenic. It is activated by a small molecular chemical inducer of dimerization that is otherwise pharmacologically inert. iCasp9 acts rapidly and has been tested in a clinical setting; graft versus host disease (GvHD) resolved after administration of the dimerization drug.[12,23]

iCasp9 is a fusion between FKBP12 with an F36V substitution and the catalytic domain of caspase 9. iCasp9 is activated by a CID, AP1903, which is a dimer of a synthetic derivative of FK506 with an ethyl substituent in place of a carbonyl group at C9.[11] The chemical substitution is complementary to the F36V amino acid substitution in FKBP12, rendering the CID non-immunosuppressive because it cannot interact with WT FKBP12. The inert pharmacology of CID was confirmed when this dimerizer was administered to normal volunteers.[24] However, AP1903 is not a marketed drug and is not widely available, greatly limiting the broad utility of iCasp9.

Suicide genes that are activated by marketed drugs have been described and used clinically. These include HSV-TK, which is activated by ganciclovir. HSV-TK, however, is highly immunogenic,[9] limiting its utility outside of clinical settings of profound immunosuppression. The RQR8[5] and huEGFRt[6] suicide genes render T cells susceptible to lysis by the therapeutic mAbs rituximab or cetuximab, respectively. These suicide genes require a sufficient local concentration of the cognate mAbs, and, consequently, activity at certain sites (e.g., behind the blood-brain barrier or in poorly vascularized tissues) may be limited. A suicide gene activated by a highly bioavailable marketed small-molecule pharmaceutical agent would be useful.

Rapamycin is an immunosuppressive drug that binds FKBP12 and the FRB domain in mTOR to inhibit mTORC1 allosterically.[25] FKBP12 serves as a cofactor in rapamycin-mediated inhibition of mTORC1. Rapamycin is well tolerated, without the renal toxicity of related agents such as tacrolimus, and was approved in 1999 for prevention of renal graft rejection.[26] Rapamycin crosses the blood-brain barrier.[27] Recently, its use has broadened to cancer treatment. Several semi-synthetic analogs, typically with derivatization at the C-43, have been developed (e.g., temsirolimus and everolimus), with improved bioavailability.[13]

Because the FRB domain is unique to TOR, rapamycin has exquisite selectivity for TOR and is effective in the nanomolar range. Rapamycin binds to FKBP12 with a high affinity (dissociation constant [K_D] = 0.2 nM); in contrast, rapamycin binds FRB with 130,000-fold lower affinity (K_D = 26 ± 0.8 μM). However, the FKBP12-Rapa complex binds FRB with a 2,000-fold higher affinity than rapamycin (12 ± 0.8 nM).[14] Typical serum levels for long-term use are 20–40 nmol,[28] although serum levels over 100 nM are readily reached using dosing of 6.5 mg/mL.[29]

We set about constructing a rapamycin-activated caspase 9. The main advantage would be a readily available dimerizing drug, licensed for commercial sale worldwide with a good biodistribution. Of note, the immunosuppressive activity of rapamycin is an advantage because, at the time of intervention, the patient suffers from too much T cell activity, and, given the very short dosing regimen needed for activation of the suicide gene, significant immune suppression would not be induced.

Physiologically, caspase 9 activates through oligomerization of its caspase activation and recruitment domain (CARD) domain via cytochrome *c*-dependent association with APAF-1. In iCasp9, the FKBP12 domain replaces the CARD domain. The first question was simply whether caspase 9 could be activated by rapamycin in an analogous way. Small-molecule AP1903 is longer than rapamycin and shows different binding kinetics to mutated FKBP12 compared with rapamycin binding to FRB and FKBP12. Efficient activation of caspase 9 with a heterodimeric, rapamycin-based system has not been previously reported. Here we demonstrate that rapamycin is highly effective at activating co-expressed FRB-Casp9/FKBP-Casp9.

This initial approach using two individual chains, each containing caspase 9, has two key drawbacks. First, there is a risk of recombination events because of the repeat of the caspase 9 gene, and second, it requires a long coding region using up space in the viral vector. Several more compact formats were constructed and tested. The format FRB-FKBP-Casp9 with a short linker between FRB and FKBP12 was optimal. Localization of FRB-FKBP12 to the C terminus led to reduced activity, perhaps because of obstruction of the catalytic site of caspase 9. Flanking the catalytic domain of FRB and FKBP12 led to lower activity and a non-flat response curve, together suggesting intramolecular bridging. Similarly, a longer linker between FRB and FBKP12 may promote the same effect.

One possible advantage of using AP1903 with the F36V-mutated FKBP12 is the lack of non-productive interactions between the suicide gene and endogenous mTOR or FKBP12. A version of rapaCasp9 was constructed where FKBP12 had the F36V substitution. Activation by CID was similar to that by rapamycin, suggesting that non-productive interactions are not limiting. Curiously, the dose response to rapamycin was flat, whereas the iCasp9 response to AP1903 could be saturated. This may be due to the higher affinity for FRB of the FKBP12-rapamycin complex than to rapamycin alone, which should prevent saturation. This is an important difference from iCasp9, whose titratability can be exploited.[30]

At 1 nM or higher of small molecule, the performance of rapaCasp9 was similar to iCasp9. As with iCasp9,[31] a gene-dose effect was observed, with a minimum threshold of transgene being needed to trigger activation regardless of the concentration of rapamycin. This phenomenon may not be very important because a low level of rapaCasp9 equals a low level of transgene. Further, this may be mitigated by more sophisticated expression systems[32,33] or, more practically, by sorting the therapeutic product for high transgene expression, which we have demonstrated.

In conclusion, we describe rapaCasp9, a rapamycin-induced caspase 9 suicide gene. Empiric structure exploration identified a format with FRB-FKBP12 fused to the catalytic domain of caspase 9, with a short linker between FRB and FKBP12 as the best configuration. Comparison with iCasp9 showed equivalent function of rapaCasp9 to iCasp9 but with the convenience of using an off-the-shelf pharmaceutical agent.

MATERIALS AND METHODS

Mice

NSG mice were obtained from Charles River Laboratories UK (under license from The Jackson Laboratory). Mice were maintained in individually ventilated cages. All animal work was performed under the United Kingdom Home Office-approved project license and in accordance with institutional policies.

Cell Culture

All cell lines and primary T cells used in the experiments were cultured in RPMI 1640 medium (Lonza) supplemented with 10% fetal bovine serum (FBS, Biosera) and 1% L-Glutamine (GlutaMAX, Gibco). Jurkat cells and SupT1 cells were purchased from the ATCC. SupT1-CD19 cells were generated by transduction with a retroviral vector encoding human CD19. T cells were generated from PBMCs obtained from National Health Service Blood and Transplant (NHSBT; Colindale, UK). Transduced T cells were cultured in the same medium as stated before, with further addition of interleukin-2 (IL-2) at 100 U/mL.

Retroviral and Plasmid Constructs

Molecular cloning was performed using a mixture of *de novo* gene synthesis of codon-optimized sequences using overlapping oligonucleotides and assembly by splicing-by-extension PCR. The sequences of all open reading frames tested are given in the Supplemental Information. Each open reading frame was cloned into the SFG retroviral transfer vector,[34] which co-expressed either EGFP or eBFP2 by cloning the fluorescent protein coding sequence downstream of the internal ribosomal entry site (IRES) from the encephalomyocarditis virus (EMCV).[35] The iCasp9 coding sequence was synthesized as described by Straathof et al.[10] The CD19-CAR used was as described by Imai et al.[17] and comprises the FMC63 scFv, a CD8 alpha stalk and trans-membrane domain along with a 41BB-CD3ζ endodomain. This was co-expressed by in-frame cloning of the foot-and-mouth 2A-like peptide from *Thosea asigna* (TaV)[18] in the order rapaCasp9-2A$_{TaV}$-CAR (termed rapaCasp9-CAR for short). A similar construct expressing iCasp9 instead of rapaCasp9 was also generated ("iCasp9-CAR"). The RQR8 sort-suicide gene was used as a control using the sequence described by Philip et al.[5] to generate the construct RQR8-CAR). The Q8 marker gene was also described by Philip et al.[5] As with RQR8, this is recognized by the QBEND/10 anti-CD34 mAb, which is used in the CD34 CliniMACS selection system. These markers were introduced into constructs with a codon-wobbled 2A peptide[18] in the configuration rapaCasp9-2A$_{TaV}$'-Q8-2A$_{TaV}$-CAR ("rapaCasp9-Q8-CAR"). Amino acid sequences of key constructs are provided in the Supplemental Information.

Transduction

The retrovirus was produced by transient transfection of 293T cells using GeneJuice (Millipore), with a plasmid encoding for gag-pol (pEQ-Pam3-E[36]), a plasmid encoding for the RD114 envelope (RDF[37]), and the desired retroviral transfer vector plasmid. Transduction was performed using Retronectin (Takara) as described previously.[5]

Flow Cytometry and Sorting

The transduction efficiency for the different suicide constructs was assessed by flow cytometry based on the expression of EGFP or eBFP2, which were included as marker genes within the plasmids used. The expression of the suicide construct co-expressed with the FMC63-CAR was determined by staining with soluble CD19 conjugated to rabbit-Fc (sCD19-Rb-Fc), followed by staining with the secondary anti-rabbit (Rb)-Fc-phycoerythrin (PE) antibody. RQR8/Q8 staining was performed using the QBEND/10 mAb. CD4, CD8, CD62L, and CD45RA staining was used for phenotypic characterization of the cells. Flow cytometry analysis was performed using the MACSQuant Analyzer 10 (Miltenyi). Flow sorting was performed using a BD FACS Melody according to the manufacturer's instructions. Magnetic cell sorting for the constructs containing Q8/RQR8 was carried out using Miltenyi hCD34 magnetic beads according to the manufacturer's protocol.

Rapamycin/AP20187-Induced Killing

Transduced cells were normalized to 50% transduction efficiency by appropriate dilution with non-transduced (NT) cells and transferred at a density of 2×10^5 cells in 96-well plates. Cells were treated with increasing concentrations (0.1–1,000 nM) of the appropriate drug, rapamycin (Sigma-Aldrich), for the rapamycin-induced suicide constructs or AP20187 (homodimerizer, Clontech Laboratories) for the iCasp9 construct. Culture medium in the absence of drug was added to the untreated cells (0 nM drug). Cells were incubated at 37°C for 24 hr before assessment of cell death by flow cytometry using Annexin V/7-AAD (BD Biosciences) staining according to the manufacturer's protocol. The expression of EGFP/eBFP2, indicating expression of the suicide constructs, after gating to the live cell population, allowed the determination of cell death within the transduced populations. The percentage of cell killing at different drug concentrations was calculated upon normalizing to the percentage of live transduced cells in the respective untreated control.

Cytotoxicity Assay

T cells expressing the FMC63-CAR (FMC63-CAR only or rapaCasp9-FMC63) were depleted of CD56-expressing cells (natural killer [NK] cells) using the EasySep human CD56 positive selection kit (STEMCELL Technologies) according to the manufacturer's instructions. Cells were then sorted into CAR^{high}-expressing cells and used in cytotoxicity assays after overnight culture. Cytotoxicity assays were set up at a 1:1 effector:target (E:T) cell ratio using 5×10^4 SupT1 WT or SupT1-CD19 targets in 96-well plates. NT cells were used in co-cultures with targets as a negative control. Co-cultures were set up in the absence of rapamycin or with 1 nM of the drug (shown

before as optimal concentration). CAR-mediated cytotoxicity was assessed by flow cytometry after 72 hr. T cells were identified from target cells by CD3 staining. 7-AAD viability dye was used for exclusion of dead cells. Viable target cells were enumerated for each co-culture condition. The percentage of remaining target cells was calculated by normalizing the number of viable target cells of each condition to that recovered from co-cultures carried out with NT T cells without rapamycin treatment (100%).

T Cell Proliferation Assay

To assess the proliferation of T cells expressing the CAR constructs in co-cultures with target cells, Cell Trace Violet staining was carried out. T cells expressing the different CAR constructs or NT T cells used as controls were labeled with Cell Trace Violet before setup of co-cultures with target cells. Labeling was performed by re-suspending the T cells at 1×10^6/mL in PBS containing Cell Trace Violet dye. Co-cultures were then set up with SupT1-NT, SupT1-CD19, Raji, and Nalm6 cells at an E:T ratio of 1:1 using 5×10^4 target cells (T cells were sorted to the transduced population). Proliferation was assessed by flow cytometry 4 days later. Cells were stained with 7-AAD and CD3 for exclusion of dead cells and detection of T cells, respectively, and the Cell Trace Violet-stained cells were used to measure proliferation by the extent of dye dilution.

In Vivo Experiments

NSG mice were injected i.v. with 2.5×10^5 Raji cells. 4 days later, the mice received a single dose of 2×10^5 of CAR T cells by tail vein injection. CAR T cells were generated by transduction of human peripheral blood T cells with either rapaCasp9-CAR or RQR8-CAR along with firefly luciferase and flow sorting for high-expressing CAR populations based on staining with sCD19. Three days after T cell infusion, the engraftment of T cells was assessed by BLI. On the same day, mice were either injected intraperitoneally (i.p.) with rapamycin at 100 μg or with carrier alone (6.25% DMSO /PBS). BLI was carried out 3 days later (day 6 after T cell injection) to assess rapamycin-induced T cell ablation. The same experimental setup as described above was used to compare rapaCasp9-CAR with iCasp9-CAR, except for this experiment, 4×10^5 sorted CAR T cells were injected per animal 4 days after Raji cell injection. 3 days later, mice injected with iCasp9-FMC63-CAR-expressing T cells were either treated with 50 μg CID (AP20187)/mouse or carrier (5% ethanol/PBS), whereas mice injected with rapaCasp9-FMC63-CAR were either treated with 5 mg/kg rapamycin or carrier (6.25% DMSO/PBS). BLI imaging and assessment of T cell ablation were carried out at the same time point as described above. Bone marrow was harvested from the animals upon termination of the study for quantification of the T cells within the bone marrow (BM) by flow cytometry. BLI for these experiments was performed using a Xenogen Vivo Vision IVIS Lumina imager. The acquisition duration for each image was 300 s (5 min).

SUPPLEMENTAL INFORMATION

Supplemental Information includes amino acid sequences and six figures and can be found with this article online at https://doi.org/10.1016/j.ymthe.2018.03.001.

AUTHOR CONTRIBUTIONS

M.S., C.G.D., and C.T.-W. performed the *in vitro* experiments. B.P. and M.S. carried out the experiments for the *in vivo* model. Experiments were designed and analyzed by M.S., S.O., S.C., S.T., and M.P. The manuscript was written by M.S. and M.P.

CONFLICTS OF INTEREST

M.P. and B.P. are inventors of a patent related to this work. All authors own stock in Autolus Ltd. M.S., C.D., C.T.-W., S.O., and S.T. are employees of Autolus Ltd. M.P. receives a salary contribution from Autolus Ltd.

ACKNOWLEDGMENTS

We wish to thank Dr. Edward Hodgkin for suggesting rapamycin to activate caspase 9 and Dr. Karin Straathof for helpful discussions and reading of this manuscript. M.P. is supported by the University College London Hospital Biomedical Research Centre.

REFERENCES

1. Morgan, R.A., Chinnasamy, N., Abate-Daga, D., Gros, A., Robbins, P.F., Zheng, Z., Dudley, M.E., Feldman, S.A., Yang, J.C., Sherry, R.M., et al. (2013). Cancer regression and neurological toxicity following anti-MAGE-A3 TCR gene therapy. J. Immunother. *36*, 133–151.
2. Morgan, R.A., Yang, J.C., Kitano, M., Dudley, M.E., Laurencot, C.M., and Rosenberg, S.A. (2010). Case report of a serious adverse event following the administration of T cells transduced with a chimeric antigen receptor recognizing ERBB2. Mol. Ther. *18*, 843–851.
3. Linette, G.P., Stadtmauer, E.A., Maus, M.V., Rapoport, A.P., Levine, B.L., Emery, L., Litzky, L., Bagg, A., Carreno, B.M., Cimino, P.J., et al. (2013). Cardiovascular toxicity and titin cross-reactivity of affinity-enhanced T cells in myeloma and melanoma. Blood *122*, 863–871.
4. Lamers, C.H.J., Sleijfer, S., Vulto, A.G., Kruit, W.H.J., Kliffen, M., Debets, R., Gratama, J.W., Stoter, G., and Oosterwijk, E. (2006). Treatment of metastatic renal cell carcinoma with autologous T-lymphocytes genetically retargeted against carbonic anhydrase IX: first clinical experience. J. Clin. Oncol. *24*, e20–e22.
5. Philip, B., Kokalaki, E., Mekkaoui, L., Thomas, S., Straathof, K., Flutter, B., Marin, V., Marafioti, T., Chakraverty, R., Linch, D., et al. (2014). A highly compact epitope-based marker/suicide gene for easier and safer T-cell therapy. Blood *124*, 1277–1287.
6. Wang, X., Chang, W.-C., Wong, C.W., Colcher, D., Sherman, M., Ostberg, J.R., Forman, S.J., Riddell, S.R., and Jensen, M.C. (2011). A transgene-encoded cell surface polypeptide for selection, in vivo tracking, and ablation of engineered cells. Blood *118*, 1255–1263.
7. Ciceri, F., Bonini, C., Stanghellini, M.T.L., Bondanza, A., Traversari, C., Salomoni, M., Turchetto, L., Colombi, S., Bernardi, M., Peccatori, J., et al. (2009). Infusion of suicide-gene-engineered donor lymphocytes after family haploidentical haemopoietic stem-cell transplantation for leukaemia (the TK007 trial): a non-randomised phase I-II study. Lancet Oncol. *10*, 489–500.
8. Straathof, K.C.M., Bollard, C.M., Popat, U., Huls, M.H., Lopez, T., Morriss, M.C., Gresik, M.V., Gee, A.P., Russell, H.V., Brenner, M.K., et al. (2005). Treatment of nasopharyngeal carcinoma with Epstein-Barr virus–specific T lymphocytes. Blood *105*, 1898–1904.
9. Berger, C., Flowers, M.E., Warren, E.H., and Riddell, S.R. (2006). Analysis of transgene-specific immune responses that limit the in vivo persistence of adoptively transferred HSV-TK-modified donor T cells after allogeneic hematopoietic cell transplantation. Blood *107*, 2294–2302.
10. Straathof, K.C., Pulè, M.A., Yotnda, P., Dotti, G., Vanin, E.F., Brenner, M.K., Heslop, H.E., Spencer, D.M., and Rooney, C.M. (2005). An inducible caspase 9 safety switch for T-cell therapy. Blood *105*, 4247–4254.
11. Clackson, T., Yang, W., Rozamus, L.W., Hatada, M., Amara, J.F., Rollins, C.T., Stevenson, L.F., Magari, S.R., Wood, S.A., Courage, N.L., et al. (1998). Redesigning

an FKBP-ligand interface to generate chemical dimerizers with novel specificity. Proc. Natl. Acad. Sci. USA *95*, 10437–10442.

12. Di Stasi, A., Tey, S.-K., Dotti, G., Fujita, Y., Kennedy-Nasser, A., Martinez, C., Straathof, K., Liu, E., Durett, A.G., Grilley, B., et al. (2011). Inducible apoptosis as a safety switch for adoptive cell therapy. N. Engl. J. Med. *365*, 1673–1683.
13. Benjamin, D., Colombi, M., Moroni, C., and Hall, M.N. (2011). Rapamycin passes the torch: a new generation of mTOR inhibitors. Nat. Rev. Drug Discov. *10*, 868–880.
14. Banaszynski, L.A., Liu, C.W., and Wandless, T.J. (2005). Characterization of the FKBP.rapamycin.FRB ternary complex. J. Am. Chem. Soc. *127*, 4715–4721.
15. Renatus, M., Stennicke, H.R., Scott, F.L., Liddington, R.C., and Salvesen, G.S. (2001). Dimer formation drives the activation of the cell death protease caspase 9. Proc. Natl. Acad. Sci. USA *98*, 14250–14255.
16. Yuan, S., Yu, X., Topf, M., Ludtke, S.J., Wang, X., and Akey, C.W. (2010). Structure of an apoptosome-procaspase-9 CARD complex. Structure *18*, 571–583.
17. Imai, C., Mihara, K., Andreansky, M., Nicholson, I.C., Pui, C.-H., Geiger, T.L., and Campana, D. (2004). Chimeric receptors with 4-1BB signaling capacity provoke potent cytotoxicity against acute lymphoblastic leukemia. Leukemia *18*, 676–684.
18. Donnelly, M.L., Hughes, L.E., Luke, G., Mendoza, H., ten Dam, E., Gani, D., and Ryan, M.D. (2001). The 'cleavage' activities of foot-and-mouth disease virus 2A site-directed mutants and naturally occurring '2A-like' sequences. J. Gen. Virol. *82*, 1027–1041.
19. Lamers, C.H., Sleijfer, S., van Steenbergen, S., van Elzakker, P., van Krimpen, B., Groot, C., Vulto, A., den Bakker, M., Oosterwijk, E., Debets, R., and Gratama, J.W. (2013). Treatment of metastatic renal cell carcinoma with CAIX CAR-engineered T cells: clinical evaluation and management of on-target toxicity. Mol. Ther. *21*, 904–912.
20. Parkhurst, M.R., Yang, J.C., Langan, R.C., Dudley, M.E., Nathan, D.-A.N., Feldman, S.A., Davis, J.L., Morgan, R.A., Merino, M.J., Sherry, R.M., et al. (2011). T cells targeting carcinoembryonic antigen can mediate regression of metastatic colorectal cancer but induce severe transient colitis. Mol. Ther. *19*, 620–626.
21. Bonifant, C.L., Jackson, H.J., Brentjens, R.J., and Curran, K.J. (2016). Toxicity and management in CAR T-cell therapy. Mol. Ther. Oncolytics *3*, 16011.
22. Marin, V., Cribioli, E., Philip, B., Tettamanti, S., Pizzitola, I., Biondi, A., Biagi, E., and Pule, M. (2012). Comparison of different suicide-gene strategies for the safety improvement of genetically manipulated T cells. Hum. Gene Ther. Methods *23*, 376–386.
23. Zhou, X., Dotti, G., Krance, R.A., Martinez, C.A., Naik, S., Kamble, R.T., Durett, A.G., Dakhova, O., Savoldo, B., Di Stasi, A., et al. (2015). Inducible caspase-9 suicide gene controls adverse effects from alloreplete T cells after haploidentical stem cell transplantation. Blood *125*, 4103–4113.
24. Iuliucci, J.D., Oliver, S.D., Morley, S., Ward, C., Ward, J., Dalgarno, D., Clackson, T., and Berger, H.J. (2001). Intravenous safety and pharmacokinetics of a novel dimerizer drug, AP1903, in healthy volunteers. J. Clin. Pharmacol. *41*, 870–879.
25. Heitman, J., Movva, N.R., and Hall, M.N. (1991). Targets for cell cycle arrest by the immunosuppressant rapamycin in yeast. Science *253*, 905–909.
26. Andoh, T.F., Burdmann, E.A., Fransechini, N., Houghton, D.C., and Bennett, W.M. (1996). Comparison of acute rapamycin nephrotoxicity with cyclosporine and FK506. Kidney Int. *50*, 1110–1117.
27. Cloughesy, T.F., Yoshimoto, K., Nghiemphu, P., Brown, K., Dang, J., Zhu, S., Hsueh, T., Chen, Y., Wang, W., Youngkin, D., et al. (2008). Antitumor activity of rapamycin in a Phase I trial for patients with recurrent PTEN-deficient glioblastoma. PLoS Med. *5*, e8.
28. Moes, D.J.A.R., Guchelaar, H.-J., and de Fijter, J.W. (2015). Sirolimus and everolimus in kidney transplantation. Drug Discov. Today *20*, 1243–1249.
29. Zimmerman, J.J., and Kahan, B.D. (1997). Pharmacokinetics of sirolimus in stable renal transplant patients after multiple oral dose administration. J. Clin. Pharmacol. *37*, 405–415.
30. Diaconu, I., Ballard, B., Zhang, M., Chen, Y., West, J., Dotti, G., and Savoldo, B. (2017). Inducible Caspase-9 Selectively Modulates the Toxicities of CD19-Specific Chimeric Antigen Receptor-Modified T Cells. Mol. Ther. *25*, 580–592.
31. Zhou, X., Naik, S., Dakhova, O., Dotti, G., Heslop, H.E., and Brenner, M.K. (2016). Serial Activation of the Inducible Caspase 9 Safety Switch After Human Stem Cell Transplantation. Mol. Ther. *24*, 823–831.
32. Kurre, P., Morris, J., Thomasson, B., Kohn, D.B., and Kiem, H.-P. (2003). Scaffold attachment region-containing retrovirus vectors improve long-term proviral expression after transplantation of GFP-modified CD34+ baboon repopulating cells. Blood *102*, 3117–3119.
33. Eyquem, J., Mansilla-Soto, J., Giavridis, T., van der Stegen, S.J.C., Hamieh, M., Cunanan, K.M., Odak, A., Gönen, M., and Sadelain, M. (2017). Targeting a CAR to the TRAC locus with CRISPR/Cas9 enhances tumour rejection. Nature *543*, 113–117.
34. Rivière, I., Brose, K., and Mulligan, R.C. (1995). Effects of retroviral vector design on expression of human adenosine deaminase in murine bone marrow transplant recipients engrafted with genetically modified cells. Proc. Natl. Acad. Sci. USA *92*, 6733–6737.
35. Bochkov, Y.A., and Palmenberg, A.C. (2006). Translational efficiency of EMCV IRES in bicistronic vectors is dependent upon IRES sequence and gene location. Biotechniques *41*, 283–284, 286, 288 passim.
36. Persons, D.A., Mehaffey, M.G., Kaleko, M., Nienhuis, A.W., and Vanin, E.F. (1998). An improved method for generating retroviral producer clones for vectors lacking a selectable marker gene. Blood Cells Mol. Dis. *24*, 167–182.
37. Cosset, F.L., Takeuchi, Y., Battini, J.L., Weiss, R.A., and Collins, M.K. (1995). High-titer packaging cells producing recombinant retroviruses resistant to human serum. J. Virol. *69*, 7430–7436.

YMTHE, Volume *26*

Supplemental Information

A Rapamycin-Activated Caspase 9-Based Suicide Gene

Maria Stavrou, Brian Philip, Charlotte Traynor-White, Christopher G. Davis, Shimobi Onuoha, Shaun Cordoba, Simon Thomas, and Martin Pule

SUPPLEMENTARY FIGURE LEGENDS

Supplementary figure 1: Co-expressed FRB-Caspase9/FKBP-Caspase9 can be activated by Rapamycin to induce cell death

(a) Jurkats transduced with the FRB-Casp9/FKBP-Casp9 and the iCasp9 constructs were treated with increasing concentrations of Rapamycin or AP20187 for the Rapamycin induced constructs and the iCasp9 respectively. Cells were incubated for 24h and then cell ablation was assessed after Annexin V/7AAD staining by flow cytometry. FACS plots show the remaining cells expressing the constructs based on the eGFP/eBFP2 marker gene expression. The percentage of killing after drug administration is shown in (b). The percentage of killing was calculated based on the percentage of remaining live cells expressing the suicide construct after drug administration and upon normalisation to the respective untreated control. Results are from 4 independent experiments (n=4). Statistical analysis was performed using repeated measures two-way ANOVA with Sidak's post-test for multiple comparisons, **** indicates the significantly higher cell killing observed in the double transduced (FRB-Casp9/FKBP-Casp9) populations as opposed to the iCasp9 transduced cells. $P<0.0001$.

Supplementary figure 2: Comparison of rapaCasp9 variants with short L1 linker and L2 linkers of either 17 or 12 amino acids.

Functions of rapaCasp9 FRB-L1:5aa-FKBP12-L2:17aa-dCasp9 vs FRB-L1:5aa-FKBP12-L2:21aa-dCasp9 were compared in primary T-cells. Peripheral blood T-cells from 3 donors were transduced with each of the 2 constructs. FKBP-Casp9 transduced cells were used as negative control. Cells were treated with increasing concentrations of Rapamycin (0-100nM) and the % of killing was assessed as before after staining with 7-AAD/Annexin V and detecting the remaining GFP+ cells within the live cell population. Statistical Analysis was carried out using 2way Anova with Tukey's multiple comparison test.

Supplementary figure 3: Testing the impact of non-productive interaction on the function of the RapaCasp9 construct

RapaCasp9 was modified to contain the mutant FKBP (F36V) as used in the iCasp9 suicide gene. This change generates a mutant rapaCasp9 responsive to the homodimerizer drug (AP20187). Jurkat cells were transduced with rapaCasp9, mutant rapaCasp9, iCasp9 or FKBP-casp9 as negative control. Transduced Jurkats were treated with increasing concentrations of Rapamycin (rapaCasp9, FKBP-Casp9) or CID (mutant rapaCasp9, iCasp). Upon 24h incubation, the drug induced cell killing was assessed with Annexin V/7AAD staining by flow cytometry. Cell death was determined based on the remaining live cells expressing the constructs, as indicated by the eGFP marker gene expression. The percentage of killing was calculated based on the percentage of remaining eGFP+ live cells after drug treatment and upon normalisation to the untreated control for each condition. (a) Representative flow data showing eGFP expression vs forward-scatter area (FSC-A) is shown; (b) Results are from 2 independent experiments (n=2).

Supplementary figure 4: Function of flow-sorted rapaCasp9-CAR T-cells

(a) RQR8-CAR and RapaCasp9-CAR constructs used for T cell transduction. The CAR used comprises of the FMC63 scFv, the CD8 stalk and transmembrane domain and a 41BB-Z endodomain. Either RQR8 or rapaCasp9 were cloned in frame upstream from the CAR separated by a foot-and-mouth disease-like 2A peptide. T-cells transduced with either of the 2 CAR constructs were sorted for CAR^{high} expression and used in co-cultures with SupT1-CD19 target cells at a ratio 1:1 E:T in the absence of Rapamycin or with the addition of Rapamycin at 1nM. After 72h incubation, the cells were stained with 7-AAD viability dye to gate to the viable cell population and CD3 for the discrimination of the T cells (CD3+) from the target cells (CD3-) and were analyzed by flow-cytometry. (b) Plots representative of a single experiment are shown. Target cell and CAR T-cell gates are indicated on the plots. (c) The percentage of viable target cells after 72h co-culture with the indicated T-cell populations is shown. Data is collective from 4 separate experiments. The percentage of remaining viable target cells was determined after normalising to the number of target cells recovered from co-cultures carried out with NT T-cells in the absence of the drug. Lines indicate the mean value of each condition for 4 separate donors. Statistical analysis was carried out using repeated measures two-way ANOVA with Sidak's post-test for multiple comparisons, ****P<0.0001.

Supplementary figure 5: Control target cell killing and Rapamycin effect after activation

(a) CD19 negative target cell control for figure 4(f) is shown. QBEND/10 sorted RapaCasp9-Q8-CAR T-cells or RQR8-CAR T-cells were incubated without Rapamycin or with increasing concentrations of Rapamycin and challenged with SupT1 cells (which are CD19 negative). (b) To test the ability of Rapamycin to kill rapaCasp9-CAR T-cells after CAR activation, RQR8-CAR or rapaCasp9-CAR T-cells were transduced and sorted. These T cells were used in co-cultures with SupT1-CD19 target cells. Co-cultures were set up at E:T ratio 1:1 with $5X10^4$ targets. Co-cultures were kept in the absence of Rapamycin for 24h-to allow the stimulation of the T cells by the targets. After 24h incubation, cells were treated with 1nM Rapamycin or were left untreated. The % of remaining T cells was assessed after FACS analysis 3d post administration of the drug. Almost complete ablation of T cells is observed within the populations expressing the rapaCasp9-CAR when they are cultured in the presence of Rapamycin. Statistical analysis was carried out using repeated measures two-way ANOVA with Sidak's post-test for multiple comparisons, ****P<0.0001.

Supplementary figure 6: Proliferation, killing and phenotype of rapaCasp9-CAR T-cells

<u>Proliferation:</u> NT T-cells or QBEND/10 sorted T-Cells expressing either RQR8-CAR or rapaCasp9-CAR were labelled with Cell Trace Violet and then used in 1:1 co-cultures with different target cell lines namely SupT1, SupT1.CD19, Raji and Nalm6 cells. Cell proliferation was assessed 4 days later by FACS analysis based on the observed dilution of the Cell Trace Violet Dye. NT T cells included as control to define the basal level of T cells proliferation. (a) Representative flow plots from one donor are shown; (b) The percentage of proliferating cells in each co-culture for the NT cells is shown. No difference between RQR8 and rapaCasp9 CAR T-cells was observed. <u>Killing:</u> NT T-cells or QBEND/10 sorted T-cells expressing either RQR8-CAR or rapaCasp9-CAR were labeled with cell trace violet and co-cultured 1:1 with either SupT1, SupT1.CD19, Raji or Nalm6 cells for 24 hours. (c) Flow-cytometric analysis of live cells corrected

for bead counting allowed the determination of surviving target cells. Cumulative data from 3 donors normalized against counts from NT effector cells are shown. There is no difference between RQR8-CAR or rapaCasp9 CAR T-cells. Phenotype: NT or sorted QBEND/10 sorted memory phenotype was determined based on the expression of CD62L and CD45RA at different time points after their transduction. (d) The memory phenotype of the transduced populations on day 7 post transduction and upon sorting was determined. (i) Cumulative data from 3 donors for CD4+ cells is shown; (ii) for CD8+ cells; (iii) shows the % of CD4+ and CD8+ cells within the transduced populations. Statistical analysis was carried out using an unpaired parametric T test and showed no difference between RQR8- and rapaCasp9-CAR T-cells.

AMINO ACID SEQUENCES

Sequences of all open reading frames (ORF) of the plasmids used in the study

>FKBP-caspase 9

```
MLEGVQVETISPGDGRTFPKRGQTCVVHYTGMLEDGKKFDSSRDRNKPFKFMLGKQEVIRGWEEGVAQMS
VGQRAKLTISPDYAYGATGHPGIIPPHATLVFDVELLKLESGGGSGVDGFGDVGALESLRGNADLAYILS
MEPCGHCLIINNVNFCRESGLRTRTGSNIDCEKLRRRFSSLHFMVEVKGDLTAKKMVLALLELAQQDHGA
LDCCVVVILSHGCQASHLQFPGAVYGTDGCPVSVEKIVNIFNGTSCPSLGGKPKLFFIQACGGEQKDHGF
EVASTSPEDESPGSNPEPDATPFQEGLRTFDQLDAISSLPTPSDIFVSYSTFPGFVSWRDPKSGSWYVET
LDDIFEQWAHSEDLQSLLLRVANAVSVKGIYKQMPGCFNFLRKKLFFKTSAS*
```

>FRB-caspase 9

```
MASRILWHEMWHEGLEEASRLYFGERNVKGMFEVLEPLHAMMERGPQTLKETSFNQAYGRDLMEAQEWCR
KYMKSGNVKDLLQAWDLYYHVFRRISKLEYSGGGSGVDGFGDVGALESLRGNADLAYILSMEPCGHCLII
NNVNFCRESGLRTRTGSNIDCEKLRRRFSSLHFMVEVKGDLTAKKMVLALLELAQQDHGALDCCVVVILS
HGCQASHLQFPGAVYGTDGCPVSVEKIVNIFNGTSCPSLGGKPKLFFIQACGGEQKDHGFEVASTSPEDE
SPGSNPEPDATPFQEGLRTFDQLDAISSLPTPSDIFVSYSTFPGFVSWRDPKSGSWYVETLDDIFEQWAH
SEDLQSLLLRVANAVSVKGIYKQMPGCFNFLRKKLFFKTSAS*
```

>rapaCasp9 (FRB-L5aa-FKBP-L17aa-caspase9)

```
MASRILWHEMWHEGLEEASRLYFGERNVKGMFEVLEPLHAMMERGPQTLKETSFNQAYGRDLMEAQEWCR
KYMKSGNVKDLLQAWDLYYHVFRRISKLEYSGGGSLEGVQVETISPGDGRTFPKRGQTCVVHYTGMLEDG
KKFDSSRDRNKPFKFMLGKQEVIRGWEEGVAQMSVGQRAKLTISPDYAYGATGHPGIIPPHATLVFDVEL
LKLESGGGGSGGGGSGGGGSGVDGFGDVGALESLRGNADLAYILSMEPCGHCLIINNVNFCRESGLRTRT
GSNIDCEKLRRRFSSLHFMVEVKGDLTAKKMVLALLELAQQDHGALDCCVVVILSHGCQASHLQFPGAVY
GTDGCPVSVEKIVNIFNGTSCPSLGGKPKLFFIQACGGEQKDHGFEVASTSPEDESPGSNPEPDATPFQE
GLRTFDQLDAISSLPTPSDIFVSYSTFPGFVSWRDPKSGSWYVETLDDIFEQWAHSEDLQSLLLRVANAV
SVKGIYKQMPGCFNFLRKKLFFKTSAS*
```

>FRB-casp9-FKBP

```
MASRILWHEMWHEGLEEASRLYFGERNVKGMFEVLEPLHAMMERGPQTLKETSFNQAYGRDLMEAQEWCR
KYMKSGNVKDLLQAWDLYYHVFRRISKLEYSGGGGSGGGGSGGGGSGVDGFGDVGALESLRGNADLAYIL
SMEPCGHCLIINNVNFCRESGLRTRTGSNIDCEKLRRRFSSLHFMVEVKGDLTAKKMVLALLELAQQDHG
ALDCCVVVILSHGCQASHLQFPGAVYGTDGCPVSVEKIVNIFNGTSCPSLGGKPKLFFIQACGGEQKDHG
FEVASTSPEDESPGSNPEPDATPFQEGLRTFDQLDAISSLPTPSDIFVSYSTFPGFVSWRDPKSGSWYVE
TLDDIFEQWAHSEDLQSLLLRVANAVSVKGIYKQMPGCFNFLRKKLFFKTSASGGGGSGGGGSGGGGSLE
GVQVETISPGDGRTFPKRGQTCVVHYTGMLEDGKKFDSSRDRNKPFKFMLGKQEVIRGWEEGVAQMSVGQ
RAKLTISPDYAYGATGHPGIIPPHATLVFDVELLKLES*
```

>FKBP-casp9-FRB

MLEGVQVETISPGDGRTFPKRGQTCVVHYTGMLEDGKKFDSSRDRNKPFKFMLGKQEVIRGWEEGVAQMS
VGQRAKLTISPDYAYGATGHPGIIPPHATLVFDVELLKLESGGGGSGGGGSGGGGSGVDGFGDVGALESL
RGNADLAYILSMEPCGHCLIINNVNFCRESGLRTRTGSNIDCEKLRRRFSSLHFMVEVKGDLTAKKMVLA
LLELAQQDHGALDCCVVVILSHGCQASHLQFPGAVYGTDGCPVSVEKIVNIFNGTSCPSLGGKPKLFFIQ
ACGGEQKDHGFEVASTSPEDESPGSNPEPDATPFQEGLRTFDQLDAISSLPTPSDIFVSYSTFPGFVSWR
DPKSGSWYVETLDDIFEQWAHSEDLQSLLLRVANAVSVKGIYKQMPGCFNFLRKKLFFKTSASGGGGSGG
GGSGGGGSMASRILWHEMWHEGLEEASRLYFGERNVKGMFEVLEPLHAMMERGPQTLKETSFNQAYGRDL
MEAQEWCRKYMKSGNVKDLLQAWDLYYHVFRRISKLEY*

>FKBP-casp9-2A-FRB-FRBw

MLEGVQVETISPGDGRTFPKRGQTCVVHYTGMLEDGKKFDSSRDRNKPFKFMLGKQEVIRGWEEGVAQMS
VGQRAKLTISPDYAYGATGHPGIIPPHATLVFDVELLKLESGGGSGVDGFGDVGALESLRGNADLAYILS
MEPCGHCLIINNVNFCRESGLRTRTGSNIDCEKLRRRFSSLHFMVEVKGDLTAKKMVLALLELAQQDHGA
LDCCVVVILSHGCQASHLQFPGAVYGTDGCPVSVEKIVNIFNGTSCPSLGGKPKLFFIQACGGEQKDHGF
EVASTSPEDESPGSNPEPDATPFQEGLRTFDQLDAISSLPTPSDIFVSYSTFPGFVSWRDPKSGSWYVET
LDDIFEQWAHSEDLQSLLLRVANAVSVKGIYKQMPGCFNFLRKKLFFKTSASQCTNYALLKLAGDVESNP
GPGVQVETISPGDGRTFPKRGQTCVVHYTGMLEDGKKVDSSRDRNKPFKFMLGKQEVIRGWEEGVAQMSV
GQRAKLTISPDYAYGATGHPGIIPPHATLVFDVELLKLESGGGGSGGGGSMLEGVQVETISPGDGRTFPK
RGQTCVVHYTGMLEDGKKFDSSRDRNKPFKFMLGKQEVIRGWEEGVAQMSVGQRAKLTISPDYAYGATGH
PGIIPPHATLVFDVELLKLES*

>Casp9-FRB-FKBP

MVDGFGDVGALESLRGNADLAYILSMEPCGHCLIINNVNFCRESGLRTRTGSNIDCEKLRRRFSSLHFMV
EVKGDLTAKKMVLALLELAQQDHGALDCCVVVILSHGCQASHLQFPGAVYGTDGCPVSVEKIVNIFNGTS
CPSLGGKPKLFFIQACGGEQKDHGFEVASTSPEDESPGSNPEPDATPFQEGLRTFDQLDAISSLPTPSDI
FVSYSTFPGFVSWRDPKSGSWYVETLDDIFEQWAHSEDLQSLLLRVANAVSVKGIYKQMPGCFNFLRKKL
FFKTSASGGGGSGGGGSGGGGSGASRILWHEMWHEGLEEASRLYFGERNVKGMFEVLEPLHAMMERGPQT
LKETSFNQAYGRDLMEAQEWCRKYMKSGNVKDLLQAWDLYYHVFRRISKLEYSGGGSLEGVQVETISPGD
GRTFPKRGQTCVVHYTGMLEDGKKFDSSRDRNKPFKFMLGKQEVIRGWEEGVAQMSVGQRAKLTISPDYA
YGATGHPGIIPPHATLVFDVELLKLESGGGG*

>iCasp9

MLEGVQVETISPGDGRTFPKRGQTCVVHYTGMLEDGKKVDSSRDRNKPFKFMLGKQEVIRGWEEGVAQMS
VGQRAKLTISPDYAYGATGHPGIIPPHATLVFDVELLKLESGGGSGVDGFGDVGALESLRGNADLAYILS
MEPCGHCLIINNVNFCRESGLRTRTGSNIDCEKLRRRFSSLHFMVEVKGDLTAKKMVLALLELAQQDHGA
LDCCVVVILSHGCQASHLQFPGAVYGTDGCPVSVEKIVNIFNGTSCPSLGGKPKLFFIQACGGEQKDHGF
EVASTSPEDESPGSNPEPDATPFQEGLRTFDQLDAISSLPTPSDIFVSYSTFPGFVSWRDPKSGSWYVET
LDDIFEQWAHSEDLQSLLLRVANAVSVKGIYKQMPGCFNFLRKKLFFKTSASRAEGRGSLLTCGDVEENP
GPMGLVRRGARAGPRMPRGWTALCLLSLLPSGFMAELPTQGTFSNVSTNVSPAKPTTTPAPRPPTPAPTI

ASQPLSLRPEACRPAAGGAVHTRGLDFACDIYIWAPLAGTCGVLLLSLVITLYCNHRNRRRVCKCPRPVV
*

>FMC63-CAR (Campana)

MGTSLLCWMALCLLGADHADACPYSNPSLCSGGGGSELPTQGTFSNVSTNVSPAKPTTTACPYSNPSLCS
GGGGSPAPRPPTPAPTIASQPLSLRPEACRPAAGGAVHTRGLDFACDIYIWAPLAGTCGVLLLSLVITLY
CNHRNRRRVCKCPRPVVRAEGRGSLLTCGDVEENPGPMETDTLLLWVLLLWVPGSTGDIQMTQTTSSLSA
SLGDRVTISCRASQDISKYLNWYQQKPDGTVKLLIYHTSRLHSGVPSRFSGSGSGTDYSLTISNLEQEDI
ATYFCQQGNTLPYTFGGGTKLEITKAGGGGSGGGGSGGGGSGGGGSEVKLQESGPGLVAPSQSLSVTCTV
SGVSLPDYGVSWIRQPPRKGLEWLGVIWGSETTYYNSALKSRLTIIKDNSKSQVFLKMNSLQTDDTAIYY
CAKHYYYGGSYAMDYWGQGTSVTVSSDPTTTPAPRPPTPAPTIASQPLSLRPEACRPAAGGAVHTRGLDF
ACDIYIWAPLAGTCGVLLLSLVITLYCKRGRKKLLYIFKQPFMRPVQTTQEEDGCSCRFPEEEEGGCELR
VKFSRSADAPAYQQGQNQLYNELNLGRREEYDVLDKRRGRDPEMGGKPRRKNPQEGLYNELQKDKMAEAY
SEIGMKGERRRGKGHDGLYQGLSTATKDTYDALHMQALPPR*

>rapaCasp9-CAR

MASRILWHEMWHEGLEEASRLYFGERNVKGMFEVLEPLHAMMERGPQTLKETSFNQAYGRDLMEAQEWCR
KYMKSGNVKDLLQAWDLYYHVFRRISKLEYSGGGSLEGVQVETISPGDGRTFPKRGQTCVVHYTGMLEDG
KKFDSSRDRNKPFKFMLGKQEVIRGWEEGVAQMSVGQRAKLTISPDYAYGATGHPGIIPPHATLVFDVEL
LKLESGGGGSGGGGSGGGGSGVDGFGDVGALESLRGNADLAYILSMEPCGHCLIINNVNFCRESGLRTRT
GSNIDCEKLRRRFSSLHFMVEVKGDLTAKKMVLALLELAQQDHGALDCCVVVILSHGCQASHLQFPGAVY
GTDGCPVSVEKIVNIFNGTSCPSLGGKPKLFFIQACGGEQKDHGFEVASTSPEDESPGSNPEPDATPFQE
GLRTFDQLDAISSLPTPSDIFVSYSTFPGFVSWRDPKSGSWYVETLDDIFEQWAHSEDLQSLLLRVANAV
SVKGIYKQMPGCFNFLRKKLFFKTSASRAEGRGSLLTCGDVEENPGPMETDTLLLWVLLLWVPGSTGDIQ
MTQTTSSLSASLGDRVTISCRASQDISKYLNWYQQKPDGTVKLLIYHTSRLHSGVPSRFSGSGSGTDYSL
TISNLEQEDIATYFCQQGNTLPYTFGGGTKLEITKAGGGGSGGGGSGGGGSGGGGSEVKLQESGPGLVAP
SQSLSVTCTVSGVSLPDYGVSWIRQPPRKGLEWLGVIWGSETTYYNSALKSRLTIIKDNSKSQVFLKMNS
LQTDDTAIYYCAKHYYYGGSYAMDYWGQGTSVTVSSDPTTTPAPRPPTPAPTIASQPLSLRPEACRPAAG
GAVHTRGLDFACDIYIWAPLAGTCGVLLLSLVITLYCKRGRKKLLYIFKQPFMRPVQTTQEEDGCSCRFP
EEEEGGCELRVKFSRSADAPAYQQGQNQLYNELNLGRREEYDVLDKRRGRDPEMGGKPRRKNPQEGLYNE
LQKDKMAEAYSEIGMKGERRRGKGHDGLYQGLSTATKDTYDALHMQALPPR*

>rapaCasp9-Q8-CAR

MASRILWHEMWHEGLEEASRLYFGERNVKGMFEVLEPLHAMMERGPQTLKETSFNQAYGRDLMEAQEWCR
KYMKSGNVKDLLQAWDLYYHVFRRISKLEYSGGGSLEGVQVETISPGDGRTFPKRGQTCVVHYTGMLEDG
KKFDSSRDRNKPFKFMLGKQEVIRGWEEGVAQMSVGQRAKLTISPDYAYGATGHPGIIPPHATLVFDVEL
LKLESGGGGSGGGGSGGGGSGVDGFGDVGALESLRGNADLAYILSMEPCGHCLIINNVNFCRESGLRTRT
GSNIDCEKLRRRFSSLHFMVEVKGDLTAKKMVLALLELAQQDHGALDCCVVVILSHGCQASHLQFPGAVY
GTDGCPVSVEKIVNIFNGTSCPSLGGKPKLFFIQACGGEQKDHGFEVASTSPEDESPGSNPEPDATPFQE
GLRTFDQLDAISSLPTPSDIFVSYSTFPGFVSWRDPKSGSWYVETLDDIFEQWAHSEDLQSLLLRVANAV
SVKGIYKQMPGCFNFLRKKLFFKTSASRAEGRGSLLTCGDVEENPGPMGLVRRGARAGPRMPRGWTALCL
LSLLPSGFMAELPTQGTFSNVSTNVSPAKPTTTPAPRPPTPAPTIASQPLSLRPEACRPAAGGAVHTRGL

```
DFACDIYIWAPLAGTCGVLLLSLVITLYCNHRNRRRVCKCPRPVVRAEGRGSLLTCGDVEENPGPMETDT
LLLWVLLLWVPGSTGDIQMTQTTSSLSASLGDRVTISCRASQDISKYLNWYQQKPDGTVKLLIYHTSRLH
SGVPSRFSGSGSGTDYSLTISNLEQEDIATYFCQQGNTLPYTFGGGTKLEITKAGGGGSGGGGSGGGGSG
GGGSEVKLQESGPGLVAPSQSLSVTCTVSGVSLPDYGVSWIRQPPRKGLEWLGVIWGSETTYYNSALKSR
LTIIKDNSKSQVFLKMNSLQTDDTAIYYCAKHYYYGGSYAMDYWGQGTSVTVSSDPTTTPAPRPPTPAPT
IASQPLSLRPEACRPAAGGAVHTRGLDFACDIYIWAPLAGTCGVLLLSLVITLYCKRGRKKLLYIFKQPF
MRPVQTTQEEDGCSCRFPEEEEGGCELRVKFSRSADAPAYQQGQNQLYNELNLGRREEYDVLDKRRGRDP
EMGGKPRRKNPQEGLYNELQKDKMAEAYSEIGMKGERRRGKGHDGLYQGLSTATKDTYDALHMQALPPR*
```

Supplementary data-Figure 1

(a)

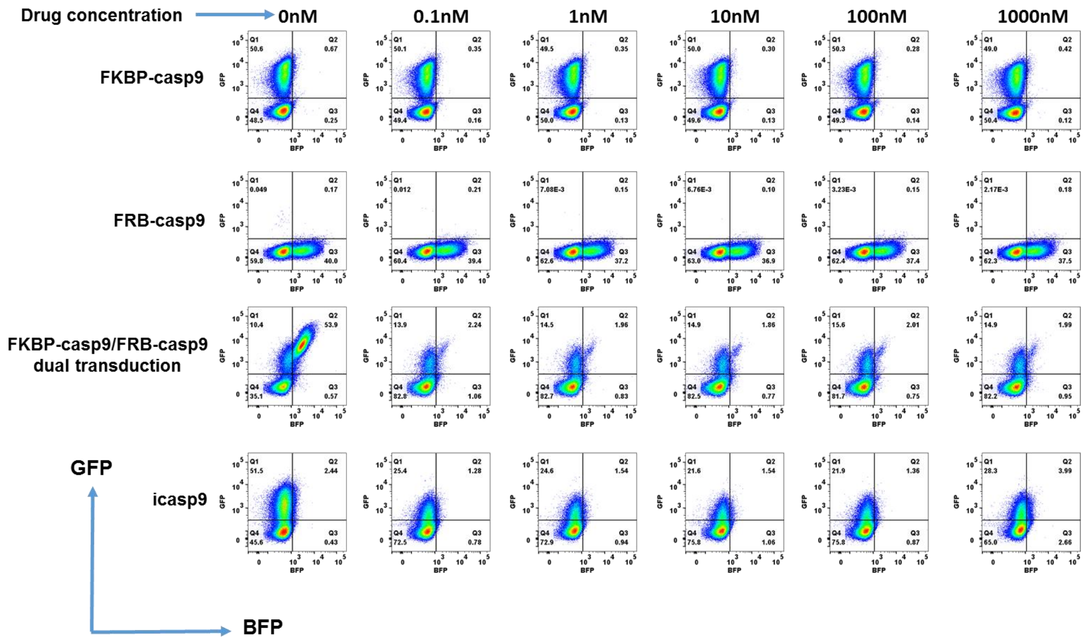

(b)

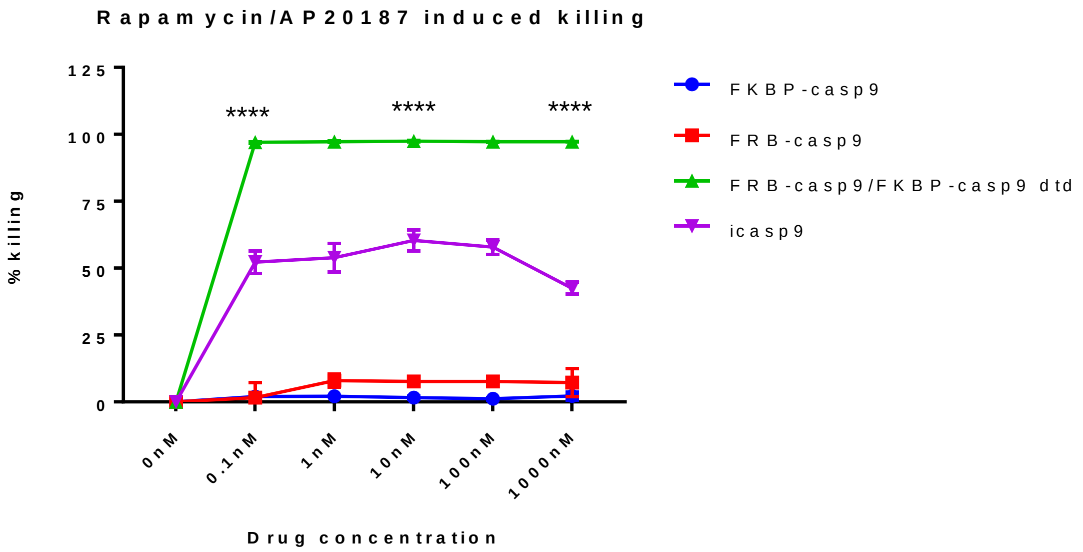

Supplementary data-Figure 2

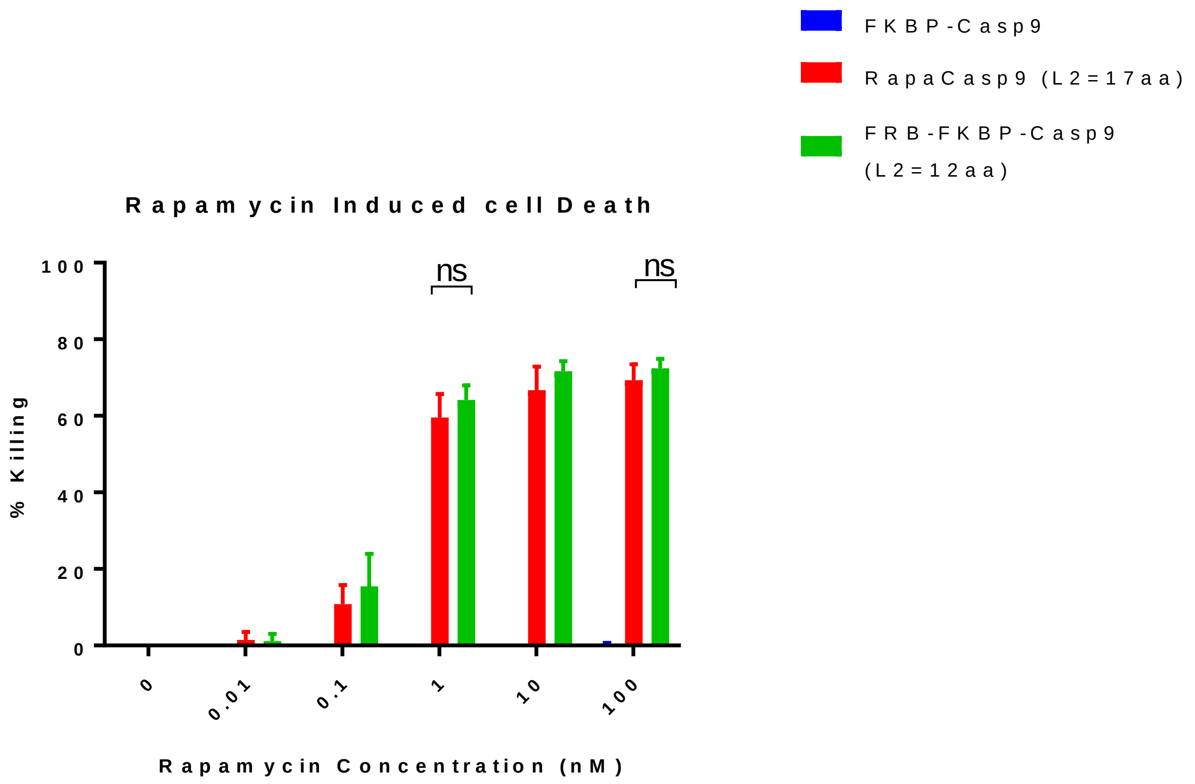

Supplementary data-Figure 3

(a)

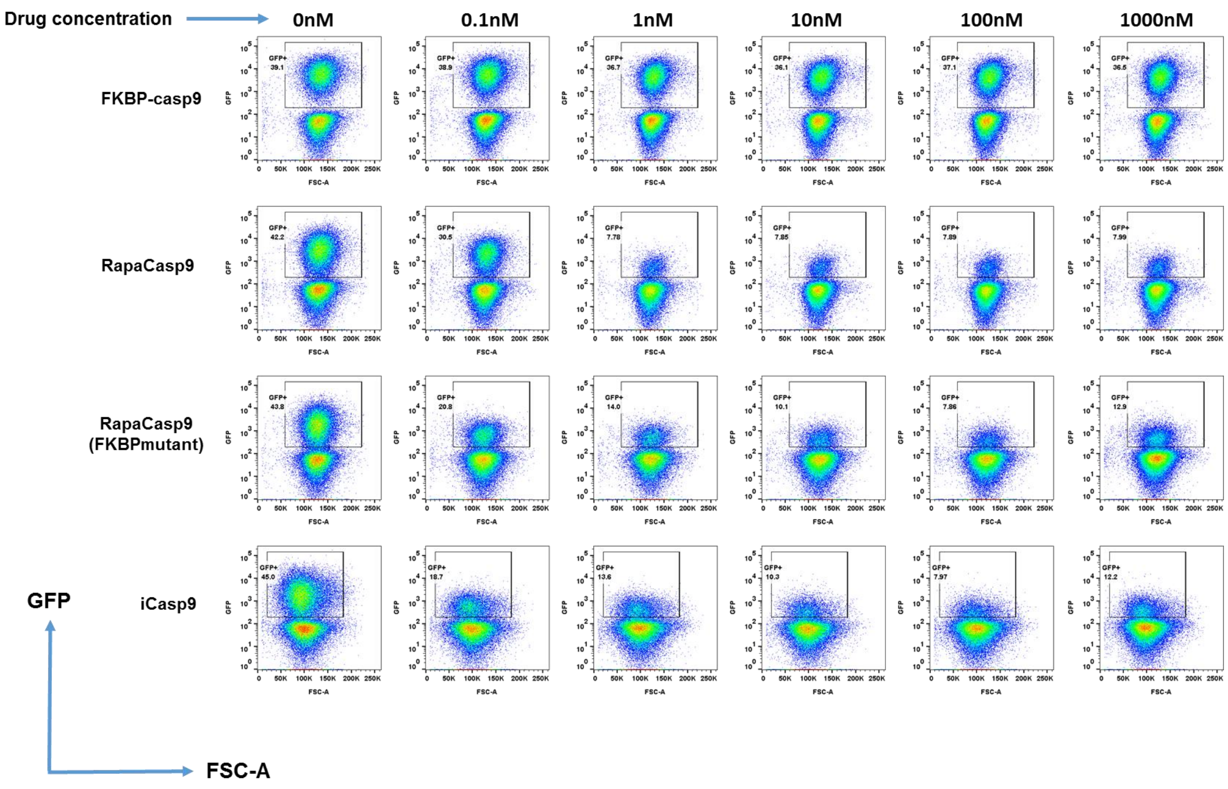

(b)

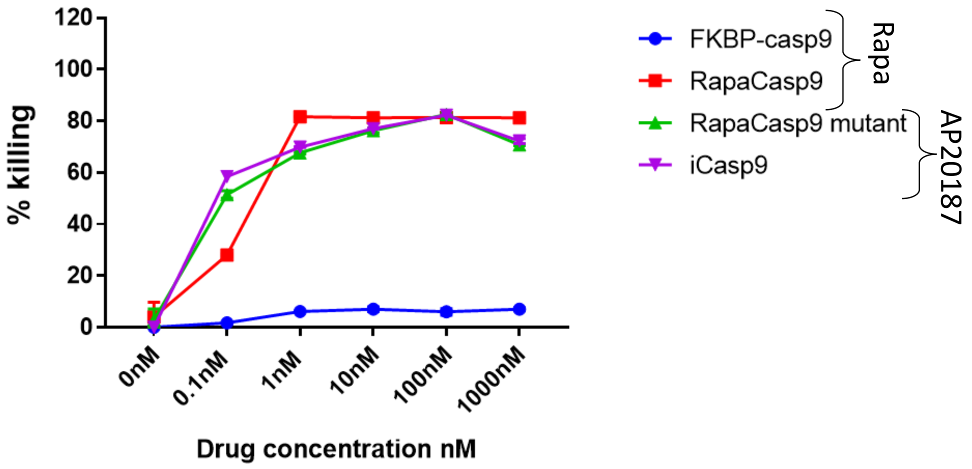

Supplementary data-Figure 4

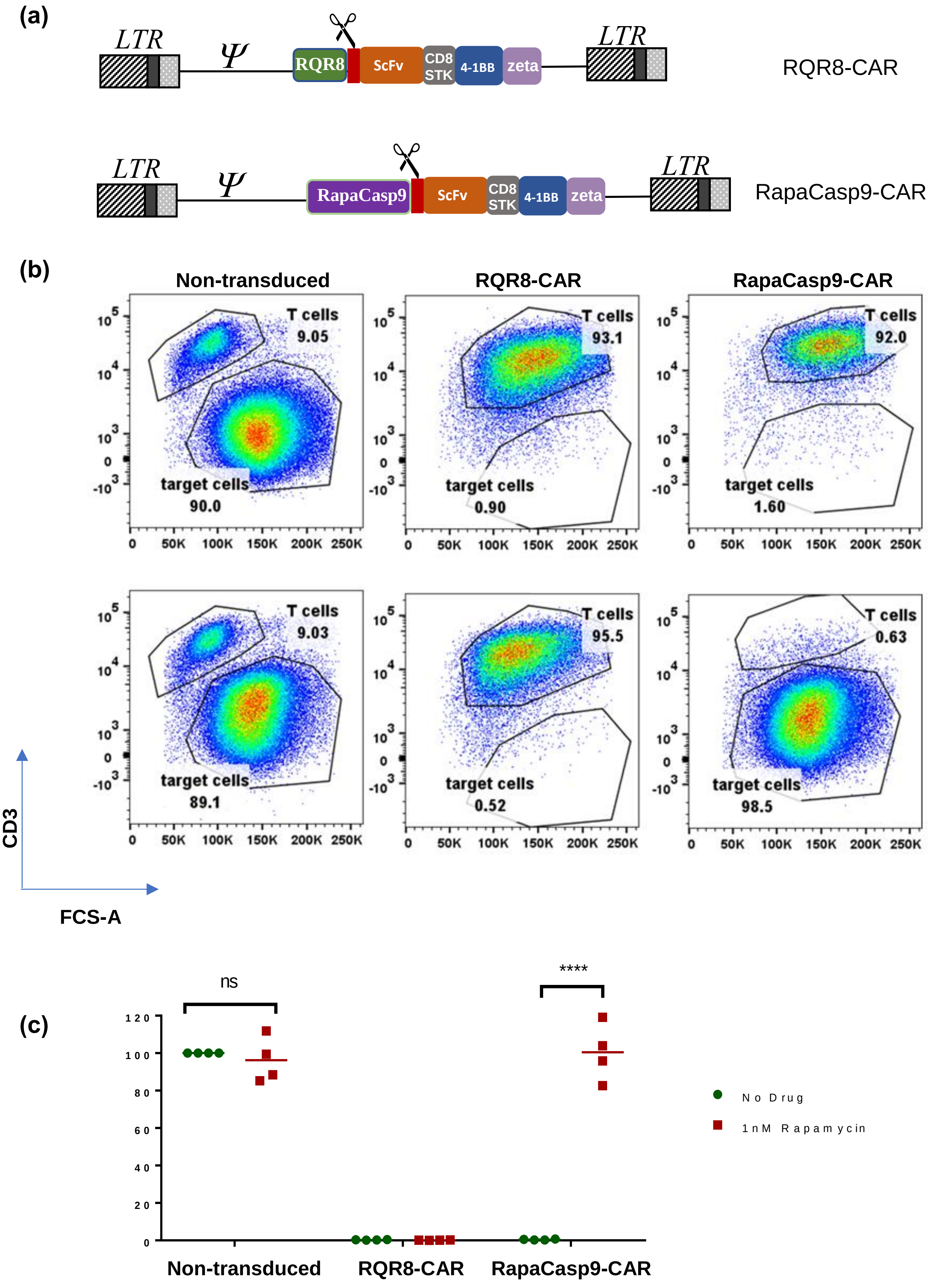

Supplementary data-Figure 5

(a)

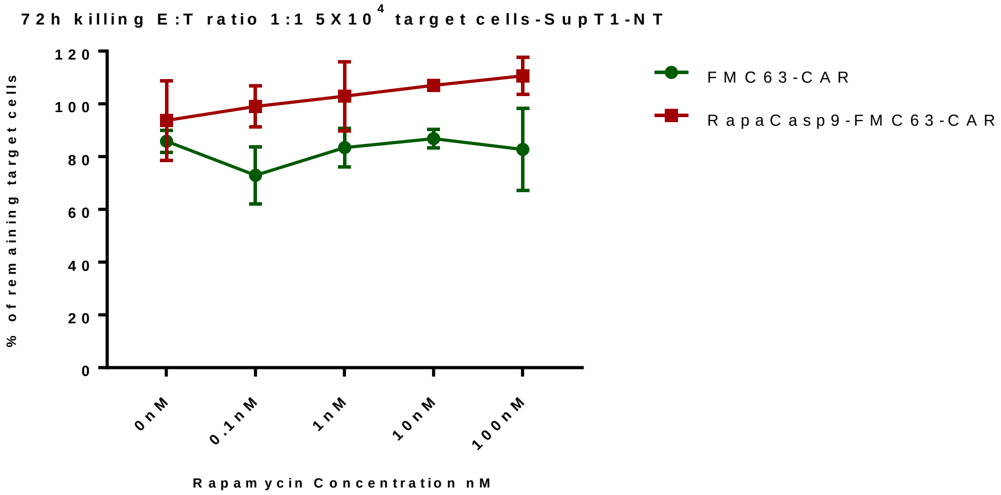

(b)

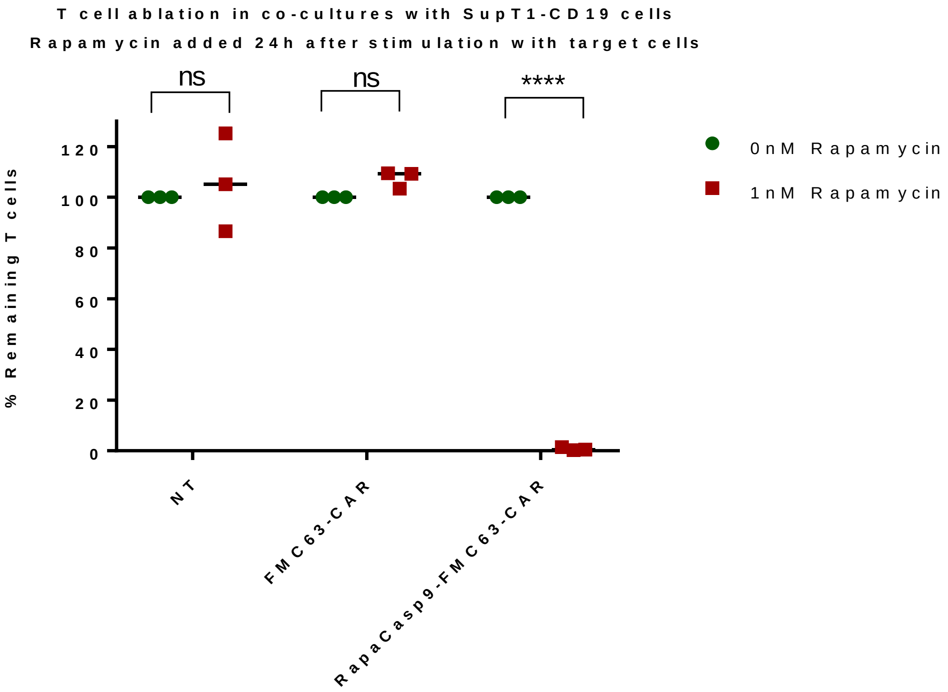

Supplementary data-Figure 6

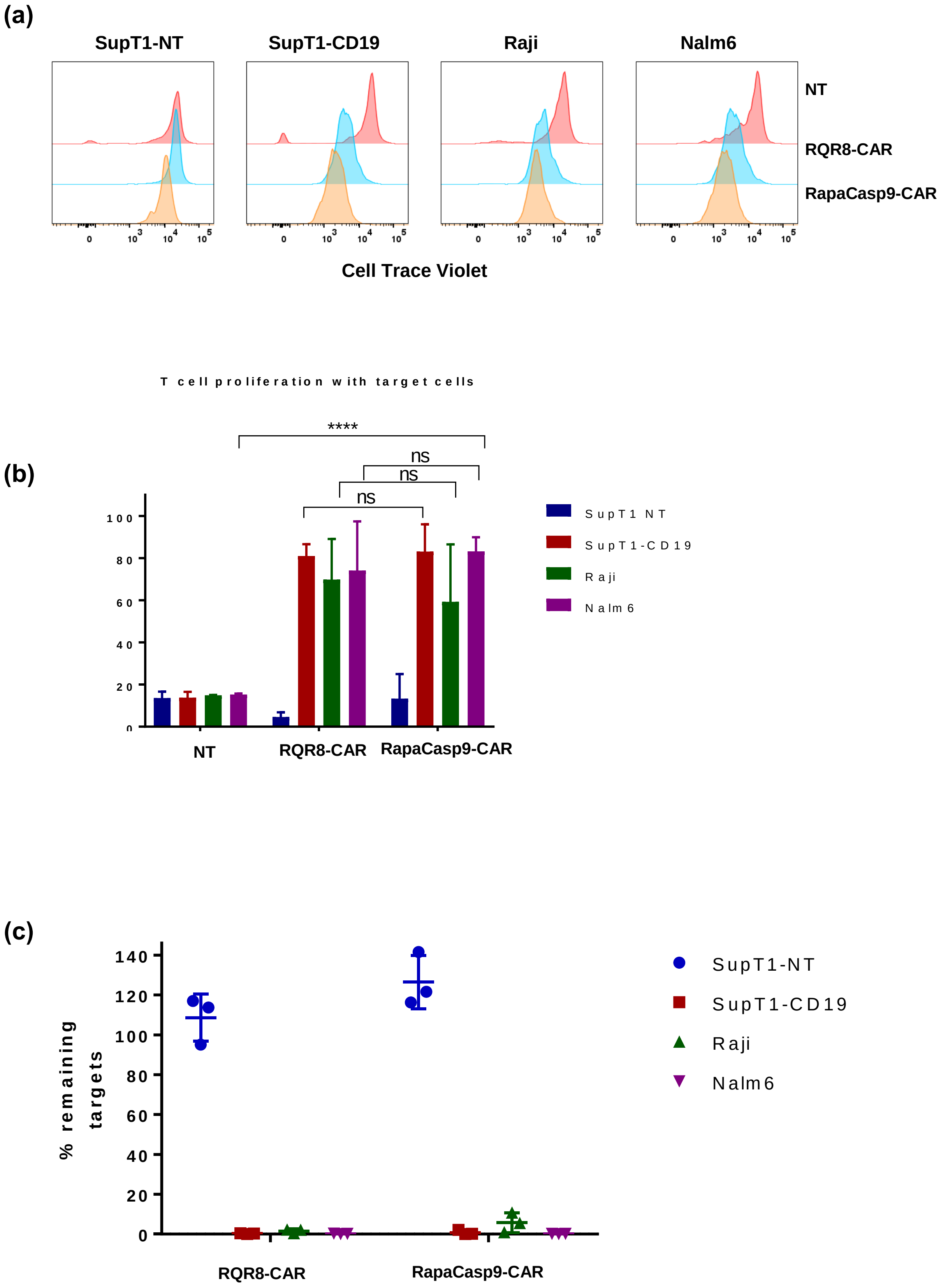

Supplementary data-Figure 6 cont'd

(d)

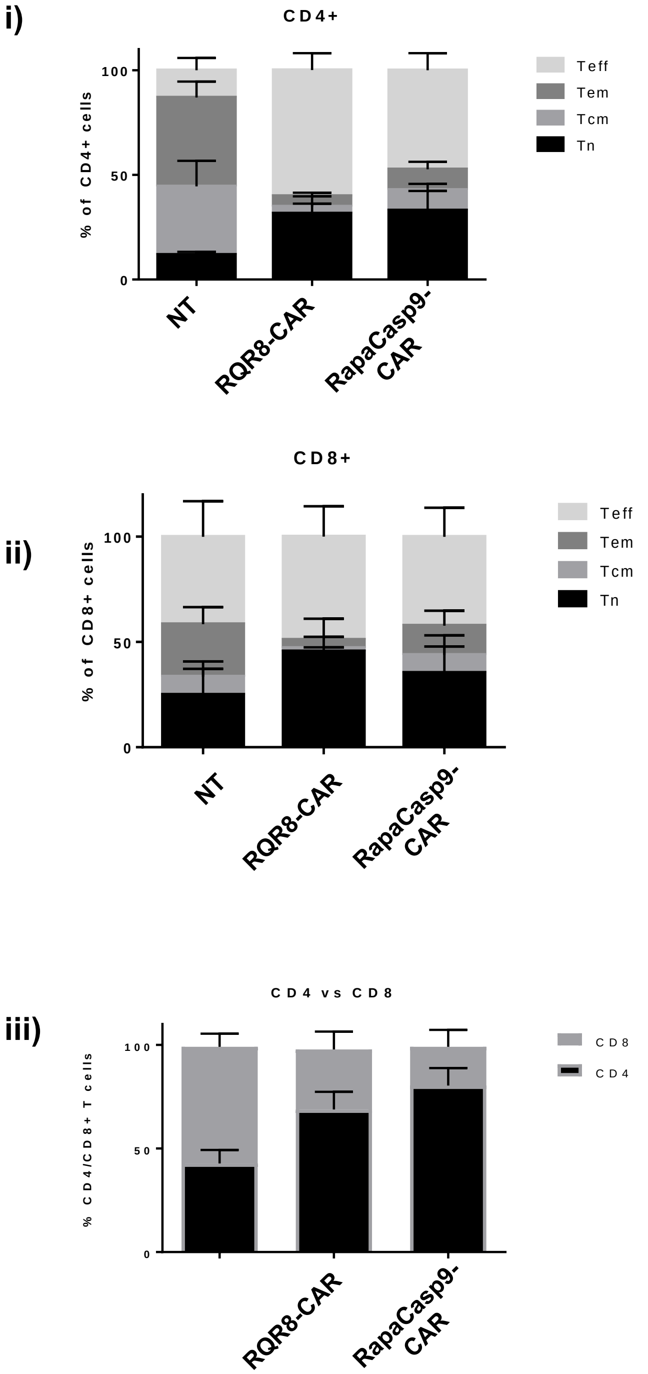

Supplement: Document S2. Article plus Supplemental Information [file mmc2.pdf]
